# Viral hijacking of host DDX60 promotes Crimean-Congo haemorrhagic fever virus replication via G-quadruplex unwinding

**DOI:** 10.1371/journal.ppat.1013278

**Published:** 2025-06-27

**Authors:** Yutong Sui, Qi Xu, Mingsheng Liu, Xiaomei Liu, Xinpeng Liu, Yujie Wang, Xiangyuan Meng, Zinan Liu, Quanshun Li, Jinyu Liu

**Affiliations:** 1 Department of Toxicology, School of Public Health, Jilin University, Changchun, China; 2 Key Laboratory for Molecular Enzymology and Engineering of Ministry of Education, School of Life Sciences, Jilin University, Changchun, China; NIAID DIR: National Institute of Allergy and Infectious Diseases Division of Intramural Research, UNITED STATES OF AMERICA

## Abstract

Crimean-Congo haemorrhagic fever virus (CCHFV) is the most prevalent tick-borne zoonotic bunyavirus, causing severe hemorrhagic fever and fatality in humans. Currently, the absence of approved vaccines or therapeutics for CCHFV infection necessitates the development of innovative therapeutic strategies. Here, we identify a guanine (G)-rich sequence located within the mRNA of the glycoprotein precursor in the medium (M) segment of the CCHFV genome, designated as M-PQS-1664(+). M-PQS-1664(+) can form stable G-quadruplex (G4) structure and functions as a negative regulatory element for viral replication. Host DDX60 is up-regulated in response to CCHFV infection, thereby it is hijacked to unwind M-PQS-1664(+) G4 for facilitating viral replication. The FDA-approved drug Cepharanthine (CEP), which competes with DDX60 to specifically stabilize M-PQS-1664(+) G4 without a global induction of host cellular G4s formation, exhibits remarkable antiviral activity *in vitro* and *in vivo*. More importantly, CEP possesses antiviral activity (50% inhibitory concentration ~ 0.2 μM) that having ~ 88 × the potency of ribavirin. Our findings underscore the CCHFV G4s as a promising target for drug development and highlight the significant potential of CEP in combating CCHFV.

## Author summary

Crimean-Congo haemorrhagic fever virus (CCHFV) was initially documented in the 1960s as a causative agent of febrile illnesses in the Congo. CCHF disease initially presents as a non-specific febrile illness, which can subsequently progress into a severe hemorrhagic condition with limited approved interventions and lacking highly efficacious treatments. Here, we describe the key roles of DDX60/RNA G-quadruplex axis in CCHFV replication. In addition, we found that FDA-approved drug Cepharanthine (CEP) stabilize the CCHFV RNA G4 by blocking DDX60 mediated G4 unwinding and exerted an excellent anti-CCHFV activity *in vitro* and *in vivo*. More importantly, our results demonstrate the potential of a combination therapy comprising Ribavirin and CEP against CCHFV. Together our data identifies DDX60/G4 axis as the potential therapeutic targets for CCHF and provides mechanistic insight into how CEP inhibits CCHFV infection.

## Introduction

Crimean-Congo Hemorrhagic fever (CCHF), which is caused by CCHF virus (CCHFV), is one of the most important vector-borne diseases of zoonotic potential, and it has been widespread in over 30 countries in Africa, Europe, Asia and the Middle East [1,2]. Worth to note, the geographical range of CCHFV continues to expand at present [3,4]. CCHF is characterized as a nonspecific febrile illness that can then progress to the severe haemorrhagic manifestations, with an average case fatality rate of 40% [5–7]. Although we have gained an improved understanding of CCHFV pathogenesis through years of efforts and enabled preclinical testing of multiple vaccine platforms and therapeutic strategies for CCHF, there are still no licensed antiviral drugs or vaccines for CCHF. And the main treatment option to manage CCHF is general supportive care. The broad-spectrum antiviral drug ribavirin has at the earliest been reported to exhibit clinical benefits for patients with CCHF, but several other studies have reported that ribavirin shows no effect on mortality rates of patients, thus its efficacy remains controversial [8–12]. Favipiravir has recently shown more significant protective effects than ribavirin in a mouse model of CCHF, but the efficacy data in patients with CCHF is limited [13]. The other potential antivirals such as 2′-Deoxy-2′-fluorocytidine [14], Molnupiravir [15] and TH3289 [16] still needed the further validation. Recently, LDLR is identified as an entry receptor for CCHFV and a soluble sLDLR-Fc fusion protein or anti-LDLR blocking antibodies can impair CCHFV infection [17,18], suggesting that the further understanding of CCHFV lifecycle contributes to novel therapeutic strategies for CCHFV infection. Given that CCHF has been listed as a priority disease by the World Health Organization (WHO) due to its great public health risk and lack of treatment options, the development of novel antiviral strategies and drugs for CCHF is an urgent need.

G-quadruplexes (G4s) are stable, non-canonical nucleic acids secondary structures adopted by guanine-rich DNAs or RNAs [19]. G4s have been shown to involve in modulating many processes of cellular biology and are viewed as potential therapeutic targets for various human disease, including cancer, neurodegenerative disorder and virosis [20–22]. Accumulating evidences indicate that G4s widely exist in the genome of multiple viruses, such as human immunodificiency virus (HIV) [23], severe acute respiratory syndrome coronavirus-2 (SARS-CoV-2) [24–26], Zika virus (ZIKV) [27,28] and Hepatitis B virus (HBV) [29,30] etc., and involve in regulating viral life cycle. G4-specific binding compounds have been used to interact with and stabilize G4s in viral genome to achieve the regulation of viral infection and replication, suggesting that they can be potential candidates as antiviral agents [31,32]. Very recently, several G4-forming sequences have been identified in CCHFV genome [33–35]. However, it remains unknown whether G4 formation occurs in the context of CCHFV infection, what roles G4s play in the CCHFV life cycle, and whether G4s can serve as therapeutic targets for CCHFV.

In this study, we identified a G-rich RNA sequence in the messenger RNA (mRNA) of CCHFV medium (M) segment, designated as M-PQS-1664(+). By performing enhanced green fluorescent protein (EGFP) and luciferase reporter gene assays, we found that M-PQS-1664(+) G4 formation inhibits the protein translation processes and host DDX60 can unwound M-PQS-1664(+) G4 to promote protein expression. In antiviral assays, DDX60 could promote recombinant CCHFV replication by targeting M-PQS-1664(+) G4. In addition, we had screened a series of G4-specific binding ligands and potential G4-stabilizing FDA-approved drugs. Cepharanthine (CEP), a biscoclaurine alkaloid widely used for the treatment of various acute and chronic diseases, stabilized M-PQS-1664(+) G4 by blocking DDX60-mediated G4 unwinding and exerted an excellent anti-CCHFV activity *in vitro* and *in vivo*. Worth to note, the anti-CCHFV activity of CEP is more potent than Ribavirin. Our findings provided a novel strategy to combat CCHFV by specifically targeting functional secondary structures within the CCHFV genome.

## Results

### Identification and annotation of G4s in CCHFV whole genome

**Fig 1:**
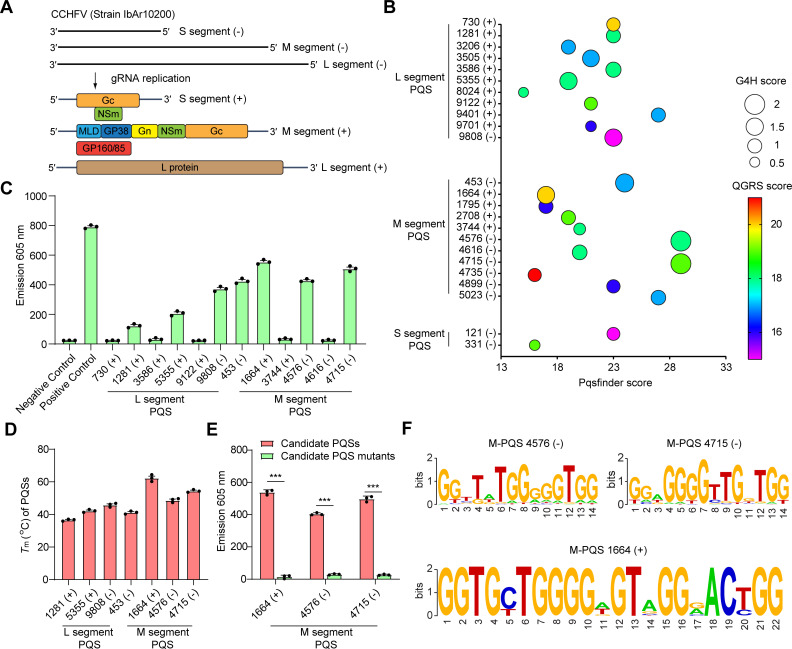
Detection and annotation of G-quadruplexes (G4s) in CCHFV genome. (A) The tri-segmented viral genome (L, M and S segments) of Crimean-Congo haemorrhagic fever virus (CCHFV) genome. L, M and S segments, large, medium and small segments. (B) CCHFV G4-forming sequences (PQSs) in the CCHFV three genomic segments. Prediction results are shown with multi-parameter analysis of Pqsfinder, G4H and QGRS scores. The (-) symbol represents CCHFV genomic RNA, while the (+) symbol denotes CCHFV mRNA. The number indicates the position of PQS within the genomic RNA of CCHFV. (C) NMM fluorescence turn-on assays for CCHFV PQS candidates. (D) *T*_m_ of CCHFV PQS candidates. (E) NMM fluorescence turn-on assays for CCHFV PQSs and their mutants. (F) The analysis of conservation of the CCHFV PQS candidates across strains. The mean of triplicate wells is represented by each point, with error bars indicating the standard error of mean (SEM). The graphs presented here are representative of three independent experiments. ^***^P < 0.001 by Student’s *t t*est.

CCHFV is an enveloped, negative-sense RNA virus that belongs to the genus *Orthonairovirus* within the family *Nairoviridae*, order *Hareavirales*, and class *Bunyaviricetes* [1,36]. The tri-segmented viral genome (Large, Medium and Small segments) of CCHFV is coated with the viral nucleoprotein (NP) and the viral proteins are encoded by three genomic segments (Fig 1A). To analyze the putative G4-forming sequences (PQSs) in CCHFV genome, we integrated multiple independent G4 prediction software and evaluated the G4 folding capabilities by Pqsfinder, G4Hunter (G4H) and QGRS scores (As described in detail in **Method**). Five PQSs in negative-sense strands and seven PQS in positive-sense strands were identified (Fig 1B), as evidenced by at least two of the cGcC, G4H, and G4NN scores exceeding the threshold (S1 Table). In addition, single strand RNA oligonucleotides of these PQSs were synthesized (S2 Table) and we used the G4-sepcific fluorescent probe N-methyl mesophorphyrin IX (NMM) and Thioflavin T (ThT) to test whether these PQS candidates can form G4 structures *in vitro* (S1A and S1B Fig). The binding of G4s to NMM and ThT can induce their fluorescence [26] and the telomeric G4 RNA and non-G4 forming RNA were used as the positive and negative control respectively. Among the 12 PQSs, we found 7 PQSs can significantly enhance the fluorescence intensity of NMM and ThT in 100 mM K^+^ buffer (Figs 1C and S1C). Furthermore, the melting temperatures (*T*_m_) of three PQSs within the M segment were all above 37 °C, suggesting that these structures are likely to maintain high stability under physiological conditions. (Fig 1D). Among the 3 PQSs within the M segment, two are located on the negative strand, specifically M-PQS-4576(-) and M-PQS-4715(-), while one is located on the positive strand, namely M-PQS-1664(+). The (-) symbol represents CCHFV genomic RNA, while the (+) symbol denotes CCHFV mRNA. The number indicates the position of PQS within the genomic RNA of CCHFV. To further confirm the G4 formation of these three PQSs, the PQS RNA oligonucleotides with G/A mutants were synthesized. Compared with that of wild type (WT) of RNA oligonucleotides, the fluorescence intensity of mutation (Mut) oligonucleotides were significantly decreased (Figs 1E and S1D). Multiple sequence alignment analysis using 357 CCHFV genomic sequences retrieved from the National Center for Biotechnology Information (NCBI) database showed that M-PQS-1664(+) was more conserved than M-PQS-4576(-) and M-PQS-4715(-) among different CCHFV strains (Fig 1F and S3 Table). Thus, we chose the M-PQS-1664(+) as representative to thoroughly investigate the behaviors of CCHFV G4.

### Identification of M-PQS-1664(+) G4 formation *in vitro*

To further verify the formation of M-PQS-1664(+) G4, we used the synthesized M-PQS-1664(+) wild type RNA oligonucleotides (M-PQS-1664(+)_WT_) and M-PQS-1664 (+) mutant RNA oligonucleotides (M-PQS-1664(+)_Mut_, with G/A mutations) to perform CD spectroscopy analysis, which is a reliable biophysical method to monitor G4 conformation. We found that M-PQS-1664(+)_WT_ forms a parallel G4 structure with a negative CD peak at 240 nm and a positive peak at 263 nm, but not for M-PQS-1664(+)_Mut_ (S2A Fig). By performing gel mobility shift assays, M-PQS-1664(+)_WT_ showed a faster migration compared with M-PQS-1664(+)_Mut_ (S2B Fig), suggesting M-PQS-1664(+)_WT_ forms a compact secondary structure. To clarify whether M-PQS-1664(+)_WT_ forms an intermolecular or intramolecular G4 structure, we performed the thermal melting assays with different concentrations of M-PQS-1664(+)_WT_ (S2C Fig). We found that the *T*_m_ of M-PQS-1664(+)_WT_ nearly unchanged with increasing M-PQS-1664(+)_WT_ concentrations, indicating that M-PQS-1664(+)_WT_ G4 is intramolecularly formed. Taken together, these results demonstrated that M-PQS-1664(+)_WT_ can form a stable parallel-type intramolecular G4 *in vitro*.

### Stabilization of M-PQS-1664(+) G4 through G4-specific ligands

**Fig 2:**
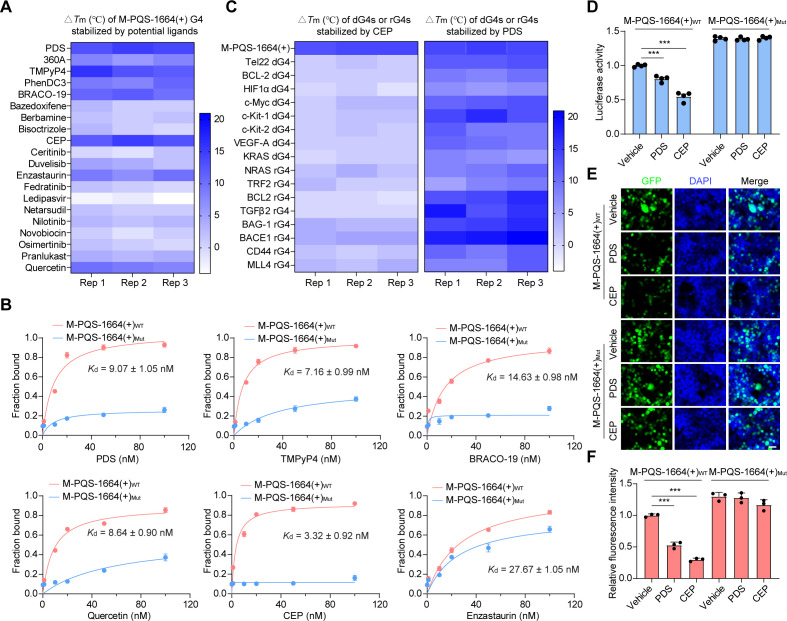
G4-specific ligands inhibits report genes expression by stabilizing CCHFV G4. The heat map of △*T*_m_ (°C) of M-PQS-1664(+) G4 stabilized by potential ligands. (B) Binding curves of compounds and M-PQS-1664(+) G4 determined by MicroScale Thermophoresis (MST) assays, and dissociation constants (*K*_d_) obtained from curve fitting (1:1) in the presence of G4. Error bars represent the SEM calculated from three replicates. (C) The heat map of △*T*_m_ (°C) of M-PQS-1664(+) G4, cellular dG4s or rG4s stabilized by CEP and PDS. (D) The luciferase activity in HEK293T cells transfected with luciferase vectors harboring M-PQS-1664(+)_WT_ or M-PQS-1664(+)_Mut_ was detected after 500 nM CEP or 500 nM PDS treatment for 24 hours by performing luciferase reporter assays. Error bars represent the SEM calculated from four replicates. (E) HEK293T cells transfected with pLV-EGFP-N vectors harboring M-PQS-1664(+)_WT_ or M-PQS-1664(+)_Mut_ were treated with 500 nM PDS or 500 nM CEP for 48 hours. Representative confocal images were demonstrated. Scale bars: 10 μm. (F) The relative fluorescent value of EGFP in transfected and treated HEK293T cells was measured. The mean of triplicate wells is represented by each point, with error bars indicating the SEM. The graphs presented here are representative of three independent experiments. ^***^P < 0.001 by Student’s *t t*est.

We subsequently conducted a screening of small molecule ligands targeting M-PQS-1664(+) G4 to elucidate its role in the life cycle of CCHFV. Alongside the continuous elucidation of G4s biological function, there has been a heightened focus on G4-specific ligands development [37]. Traditional G4-specific ligands, including pyridostatin (PDS) and TMPyP4 etc., have been utilized to target viral G4 structures [24,32]. However, these compounds generally exhibit suboptimal drug-like properties and selectivity profiles, thereby constraining their clinical applicability. We next evaluated the interaction ability of both traditional G4-specific ligands (S3A Fig) and a series of potential G4-stabilizing FDA-approved drugs (S3B Fig) with M-PQS-1664(+) G4. By performing melting assays, we found that traditional G4-specific ligands PDS, TMPyP4 and BRACO-19 and potential G4-stabilizing FDA-approved drugs Quercetin, Cepharanthine (CEP) and Enzastaurin show powerful activity for enhancing M-PQS-1664(+) G4 stability (Fig 2A). Further, we analyzed the binding constant of these candidate compounds with M-PQS-1664(+) G4 by performing MicroScale Thermophoresis (MST) assays. The results showed that M-PQS-1664(+) G4 binds strongly with five candidate compounds, with the *K*_d_ values being 9.07 ± 1.05, 7.16 ± 0.99, 14.63 ± 0.98, 8.64 ± 0.90 and 3.32 ± 0.92 nM for PDS, TMPyP4, BRACO-19, Quercetin and CEP, respectively (Fig 2B). PDS and CEP were selected as representative compounds for subsequent G4 functional studies. Worth to note, CEP, but not PDS, preferred to stabilize M-PQS-1664(+) G4 rather than other cellular DNA or RNA G4s (Fig 2C), suggesting the specificity and selectivity of CEP among different G4s.

### Repression of reporter gene expression through M-PQS-1664(+) G4 stabilization

We next investigated the biological function of M-PQS-1664(+) G4 by using PDS and CEP. M-PQS-1664(+) G4 is located in mRNA of CCHFV M segment, which encodes the viral glycoprotein precursor (GPC). Given that G4s formed in mRNA participate in regulation of translation-related processes [38], we speculated M-PQS-1664(+) G4 may affect the viral GPC expression. To this end, we conducted enhanced green fluorescent protein (EGFP) and luciferase reporter gene systems. The M-PQS-1664(+)_WT_ and M-PQS-1664(+)_Mut_ sequences were cloned into the EGFP and luciferase vectors (S4 Fig). We found that PDS and CEP treatment significantly inhibit the EGPF expression and luciferase activity in the HEK293T cells transfected with EGFP and luciferase vectors harboring M-PQS-1664(+)_WT_ sequences, but not that harboring M-PQS-1664(+)_Mut_ (Figs 2D-F and S4A-S4F), suggesting that stabilizing M-PQS-1664(+)_WT_ G4 by PDS and CEP may inhibit mRNA translation. In addition, we generated a recombinant CCHFV expressing a reporter protein as previously described reverse genetics system [14], allowing us to quantify viral load by measuring the ZsGreen1 (ZsG) fluorescence in infected cells (S5A and S5B Fig). The recombinant CCHFV features a modified S genome segment wherein the ZsG coding sequence is inserted upstream of the NP coding sequence, separated by the porcine teschovirus-1 2A peptide linker sequence (P2A) [39]. ZsG has previously demonstrated robust fluorescence levels in analogous recombinant viral reporter assays [40]. We found that PDS and CEP treatment lead to a significant reduction of ZsG fluorescence in recombinant CCHFV-infected Huh7, SW13 and Vero E6 cells (S5C Fig). Meanwhile, there was no significant cytotoxicity observed in the HEK293T, Huh7, SW13 and Vero E6 cells treated with PDS and CEP at the effective concentration (S6A and S6B Fig). Collectively, these results suggested that M-PQS-1664(+) G4 stabilization by PDS and CEP inhibits reporter gene expression and recombinant CCHFV replication.

### Host DDX60 binds and unwinds M-PQS-1664(+) G4

**Fig 3:**
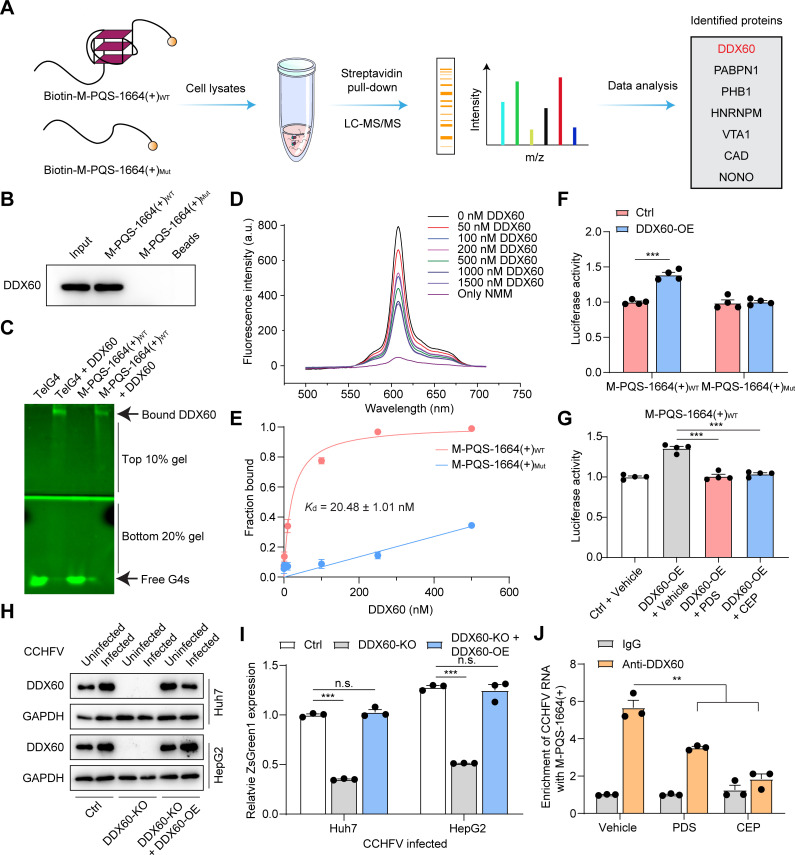
DDX60 promotes CCHFV infection by unfolding G4. Mass spectrometry representation for RNA pull-down from Huh7 cell lysates using M-PQS-1664(+) G4 and its mutated counterparts. A list of enriched proteins is mentioned showing interaction (Figure was modiﬁed from https://openclipart.org/.). (B) RNA pull-down of M-PQS-1664(+) G4, and their mutated counterparts followed by western blotting assays to detect DDX60 protein. (C) Electrophoretic mobility shift assay (EMSA) results show the binding of DDX60 to M-PQS-1664(+) G4 and Telomere G4 (TelG4). (D) DDX60 can unfold M-PQS-1664(+) G4 in concentration-dependent manners in the presence of 1 mM ATP. (E) Binding curves of DDX60 and M-PQS-1664(+) G4 determined by MST assays, and dissociation constants (*K*_d_) obtained from curve fitting (1:1) in the presence of G4. Error bars represent the SEM calculated from three replicates. (F) The activity luciferase in DDX60-OE HEK293T cells transfected with luciferase vectors harboring M-PQS-1664(+) G4_WT_ or M-PQS-1664(+) G4_Mut_ was detected by performing luciferase reporter assays. DDX60 overexpression, DDX60-OE. Error bars represent the SEM calculated from four replicates. (G) 500 nM CEP and 500 nM PDS treatment for 24 hours inhibit DDX60 mediated increase of luciferase activity. Error bars represent the SEM calculated from four replicates. (H and I) The protein levels of DDX60 in DDX60 WT (Ctrl) and DDX60 knockout (DDX60-KO) Huh7 and HepG2 cells as well as DDX60-KO Huh7 and HepG2 cells transiently transfecting DDX60. The cells were infected without or with CCHFV/ZsG at MOI 0.1., subsequently the protein levels of DDX60 (H) were detected at 72 hours post infection by western blotting assays. The ZsG fluorescence (green) was determined at 72 hours post infection (I). DDX60 knockout, DDX60-KO. (J) 500 nM PDS or 500 nM CEP treatment inhibit the interaction between DDX60 and the CCHFV RNA containing M-PQS-1664(+) in the recombinant CCHFV-infected Huh7 cells. The cells were infected with CCHFV/ZsG at MOI 0.1., and the enrichment of CCHFV RNA containing M-PQS-1664(+) by anti-DDX60 was determined at 48 hours post infection by performing RNA immunoprecipitation (RIP) assays. The mean of triplicate wells is represented by each point, with error bars indicating the SEM. The graphs presented here are representative of three independent experiments. ^**^P < 0.01, ^***^P < 0.001 by Student’s *t* tes*t*.

Considering the inhibitory effects of M-PQS-1664(+) G4 on CCHFV, it is hypothesized that certain viral or host proteins may play crucial roles in regulating the folding/unfolding dynamics of M-PQS-1664(+) G4, thereby participating in regulation of CCHFV life cycle. We therefore performed affinity enrichment methods to identify the M-PQS-1664(+) G4 binding proteins (Fig 3A). Biotinylated oligonucleotides containing the M-PQS-1664(+) G4 sequences were folded into RNA G4 structure, and folded M-PQS-1664(+) G4 and control oligonucleotides were immobilized on streptavidin beads and used as baits for affinity enrichments of proteins from Huh7 cell cytosolic extracts. Next, proteins bound to the M-PQS-1664(+) G4 oligonucleotides and control samples (M-PQS-1664(+)_Mut_ G4 and empty beads) were subjected to on-bead tryptic digestion followed by LC-MS/MS. Mass spectrometry analyses revealed that the most abundant protein associated with M-PQS-1664(+) G4 is RNA helicases DDX60 (Fig 3A). DDX60 belongs to DExH helicase family that is generally responsible for unfolding the G4 structure [41]. First, the association of DDX60 with M-PQS-1664(+) G4 was validated by western blotting assays (Fig 3B). By performing electrophoretic mobility shift assay (EMSA), we found that DDX60 can directly bind to M-PQS-1664(+) G4 and telomere G4 (as a positive control) (Fig 3C). Fluorescence turn-on assays by using NMM showed that DDX60 possesses the ability to unfold M-PQS-1664(+) G4 in concentration-dependent manners in the presence of ATP (Fig 3D). Furthermore, by performing MST assays in the absence of ATP, our analysis revealed a robust binding affinity between DDX60 and M-PQS-1664(+) G4, as evidenced by the *K*_d_ values of 20.48 ± 1.01 nM (Fig 3E). More importantly, we found that DDX60 overexpression enhances luciferase activity in HEK293T cells transfected with luciferase vectors harboring M-PQS-1664(+)_WT_ sequences, but not that harboring M-PQS-1664(+)_Mut_ (Fig 3F). In addition, CEP and PDS treatment could inhibit the DDX60 mediated enhancement of luciferase activity (Fig 3G), suggesting that CEP and PDS may block DDX60 mediated M-PQS-1664(+) G4 unwinding.

A recent study showed that CCHFV infection induces the increase of DDX60 mRNA levels [42]. Indeed, by conducting data mining on RNA-seq data (accession number GSE133086) of CCHFV infection, we identified a significant up-regulation of DDX60 mRNA levels following CCHFV infection (S7A and S7B Fig, S4 and S5 Tables). Consistently, the protein levels of DDX60 were significantly increased in the recombinant CCHFV-infected HepG2 and Huh7 cells (Fig 3H). Therefore, it is postulated that the up-regulation of DDX60 expression during CCHFV infection potentially facilitates CCHFV replication by unwinding M-PQS-1664(+) G4. Indeed, CRISPR-Cas9 mediated DDX60 knockout caused a significant reduction of ZsG fluorescence in recombinant CCHFV-infected Huh7 and HepG2 cells, whereas DDX60 overexpression enhanced the ZsG fluorescence in DDX60 knockout Huh7 and HepG2 cells (Fig 3H and 3I), suggesting that DDX60 can promote CCHFV replication. Consistently, DDX60 knockout decreased the protein levels of viral glycoprotein, while DDX60 overexpression increased viral glycoprotein expression (S7C Fig). Moreover, the results of CCK-8 assays indicated that DDX60 knockdown has no significant effect on the viability of HepG2 and Huh7 cells (S7D and S7E Fig). In agreement with the *in vitro* interaction experiments, RNA immunoprecipitation (RIP) assays demonstrated that DDX60 directly interacts with the CCHFV RNA containing M-PQS-1664(+) in recombinant CCHFV-infected Huh7 cells (Fig 3J). We found that PDS and CEP can significantly inhibit the interaction between DDX60 and the CCHFV RNA containing M-PQS-1664(+) (Fig 3J). Moreover, PDS and CEP inhibited DDX60-mediated promotion of CCHFV replication (S8 Fig). Meanwhile, we observed that PDS and CEP can still inhibit CCHFV replication in DDX60-knockout Huh7 cells (S8 Fig). These findings demonstrated that CCHFV may hijack host DDX60 to unwind M-PQS-1664(+) G4, thereby facilitating viral replication. This process could be targeted by PDS and CEP as potential strategy to combat CCHFV.

### Antiviral results of G4-specific ligands in cells

**Fig 4:**
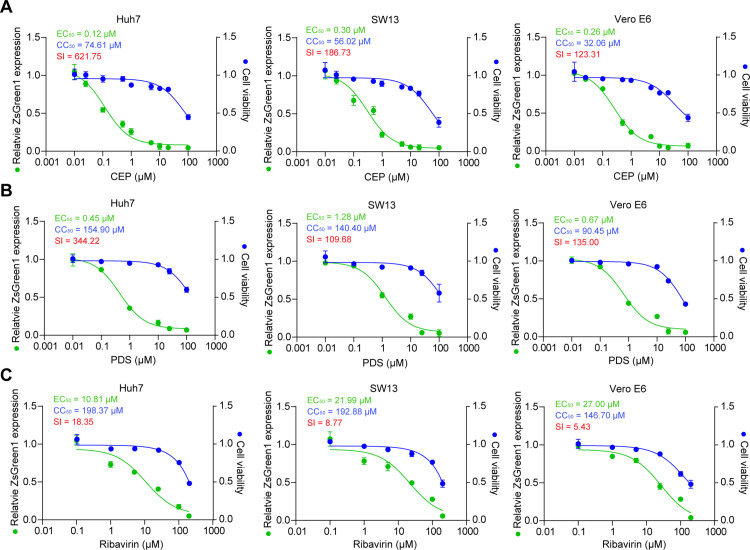
Anti-CCHFV activity of candidate drugs in cell-based assays. (A-C) Dose-response curves were generated in Huh7, SW13, or Vero E6 cells following treatment with varying concentrations of CEP (A), PDS (B) or Ribavirin (C) prior to infection with CCHFV/ZsG at MOI 0.1. The reduction in ZsG fluorescence (green) was determined at 72 hours post infection. Cell viability was determined using the same concentrations of compounds. The mean of triplicate wells is represented by each point, with error bars indicating the SEM. The graphs presented here are representative of three independent experiments. EC_50_, half-maximal effective doses. CC_50_, half-cytotoxic concentration. SI, selectivity index.

We next evaluated whether PDS and CEP can be used as potential anti-CCHFV drugs. Standard assays were carried out to measure the effects of PDS, CEP and Ribavirin (as a positive control) on the cytotoxicity and fluorescence intensity in Huh7, SW13 and monkey kidney Vero E6 cells. The half-cytotoxic concentration (CC_50_) of these compounds was determined to be 74.61 μM, 56.02 μM and 32.06 μM for CEP, 154.90 μM, 140.40 μM and 90.45 μM for PDS and 198.37 μM, 192.88 μM and 146.70 μM for Ribavirin in Huh7, SW13 and Vero E6 cells, respectively (Fig 4A-C). In addition, half-maximal effective doses (EC_50_) and selectivity index (SI) of the three compounds were determined. Compared with PDS (EC_50_ = 0.45 μM, 1.28 μM and 0.67 μM; SI = 344.22, 109.68 and 135.00 in Huh7, SW13 and Vero E6 cells, respectively) and Ribavirin (EC_50_ = 10.81 μM, 21.99 μM and 27.00 μM; SI = 18.35, 8.77 and 5.43 in Huh7, SW13 and Vero E6 cells, respectively), CEP (EC_50_ = 0.12 μM, 0.30 μM and 0.26 μM; SI = 621.75, 186.73 and 123.31 in Huh7, SW13 and Vero E6 cells, respectively) exhibited higher anti-CCHFV activity and a higher SI (Fig 4A-C). Time-of-addition assays suggested that CEP and PDS function at a stage post CCHFV entry (S9A-S9C Fig), consistent with its putative antiviral mechanism by targeting viral G4s.

**Fig 5:**
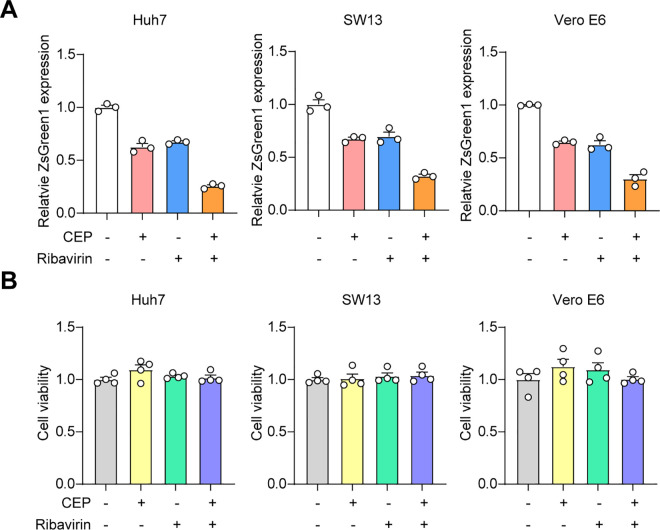
CEP combined with ribavirin inhibits CCHFV infection. (A) The Huh7, SW13, and Vero E6 cells were treated with the indicated compounds either individually or in combination at the following concentrations: CEP (400 nM) and ribavirin (10 μM). Subsequently, the cells were infected with CCHFV/ZsG at MOI 0.1. The reduction in ZsG fluorescence (green) was determined at 72 hours post infection. Each data point represents the mean value obtained from triplicate wells, with error bars indicating standard deviation. (B) Cell viability was determined concurrently using the indicated compounds in Huh7, SW13, and Vero E6 cells. Each data point represents the mean value obtained from quadruplicate wells, with error bars indicating SEM.

Given the standard use of Ribavirin in CCHF treatment, we next investigated whether Ribavirin would synergize with CEP or PDS. We found that the combination of Ribavirin and CEP or PDS exhibited a more effective anti-CCHFV effect compared to using either drug alone (Figs 5A and S10A). And cell viability was determined simultaneously for all combinations tested, and no cytotoxicity was observed (Figs 5B and S10B). These findings demonstrate the potential of a combination therapy comprising Ribavirin and CEP for the treatment of CCHF.

### CEP and PDS inhibit CCHFV infection by stabilizing M-PQS-1664(+) G4

**Fig 6:**
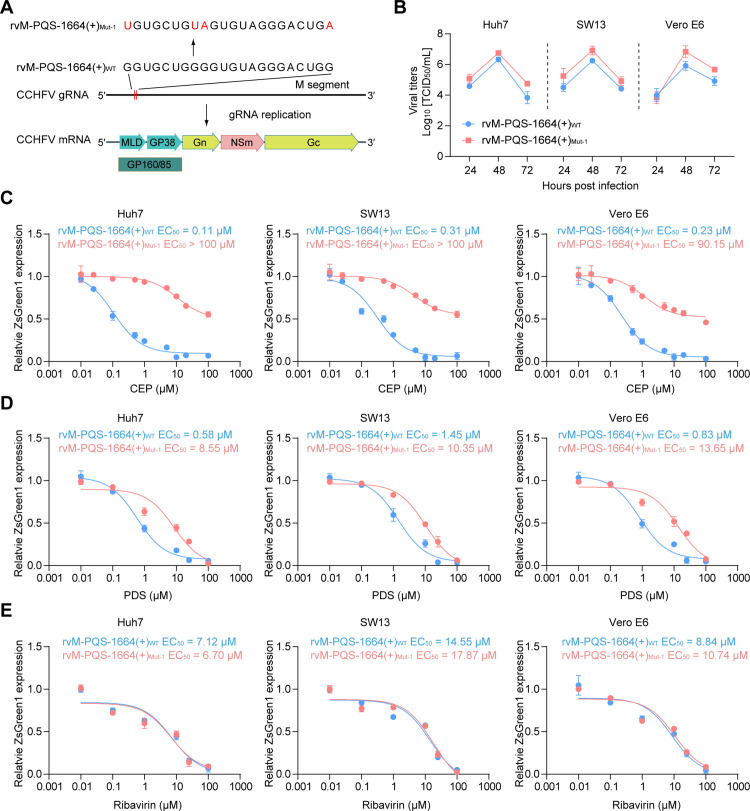
CEP and PDS inhibit CCHFV infection by targeting G4. Schematic representation of G4-disruptive mutations in the CCHFV genome. (B) Viral growth curves in Huh7, SW13, and Vero E6 cells infected with either rvM-PQS-1664(+)_WT_ or rvM-PQS-1664(+)_Mut-1_ at MOI 0.1. Viral titers were determined at 24, 48, and 72 hours post infection. rvM-PQS-1664(+)_WT_, recombinant virus with M-PQS-1664(+)_WT._ rvM-PQS-1664(+)_Mut-1_, recombinant virus with M-PQS-1664(+)_Mut-1_. (C-E) Dose-response curves were generated in Huh7, SW13, or Vero E6 cells following treatment with varying concentrations of CEP (C), PDS (D) or Ribavirin (E) prior to infection with rvM-PQS-1664(+)_WT_ or rvM-PQS-1664(+)_Mut-1_ at MOI 0.1. The reduction in ZsG fluorescence (green) was determined at 72 hours post infection. The mean of triplicate wells is represented by each point, with error bars indicating the SEM. The graphs presented here are representative of three independent experiments.

To further confirm the contribution of M-PQS-1664(+) G4 to CEP and PDS mediated antiviral activity, we constructed the recombinant CCHFV with the G4-disruptive mutation (recombinant virus with M-PQS-1664(+)_Mut-1_, rvM-PQS-1664(+)_Mut-1_). By referencing the multiple sequence alignment results, we mutated partial G to other bases that occurred in other CCHFV strains in the corresponding sites for destroying the G4 structure (Fig 6A). Fluorescence turn-on assays by using NMM and ThT showed that the partial G mutant within M-PQS-1664(+) lead to complete destruction of the G4 structure (S11A and S11B Fig). Consistently, we found that PDS and CEP treatment cannot affect luciferase activity in the HEK293T cells transfected with luciferase vectors harboring M-PQS-1664(+)_Mut-1_ (S11C Fig).

To establish the suitability of rvM-PQS-1664(+)_Mut-1_ for studying CCHFV replication, we examined its growth kinetics in 3 human cell lines: Huh7, SW13 and Vero E6 cells. These cell lines were infected with either rvM-PQS-1664(+)_WT_ or rvM-PQS-1664(+)_Mut-1_ at MOI 0.1, and titers (TCID_50_/mL) were determined at 24, 48, and 72 hours post infection. In all cell lines tested, the viral titers of rvM-PQS-1664(+)_WT_ were slightly lower than those of rvM-PQS-1664(+)_Mut-1_ and peaked at 48 hours post infection (Fig 6B). The increased viral replication of rvM-PQS-1664(+)_Mut-1_ might be attributed to M-PQS-1664(+) G4-disruptive mutation, which promotes viral protein expression.

Indeed, the protein levels of viral glycoprotein in the rvM-PQS-1664(+)_Mut-1_ infected DDX60-KO Huh7 cells were higher than those in the rvM-PQS-1664(+)_WT_ infected cells (S12A Fig). Consistently, RIP assays indicated that DDX60 cannot interact with the CCHFV RNA harboring M-PQS-1664(+)_Mut-1_ (S12B Fig), and the rvM-PQS-1664(+)_Mut_ exhibited a significantly reduced sensitivity to DDX60 knockout compared with rvM-PQS-1664(+)_WT_ (S12C and S12D Fig). In addition, we detected the antiviral activity of PDS, CEP and Ribavirin against rvM-PQS-1664(+)_WT_ and rvM-PQS-1664(+)_Mut-1_. Infection with rvM-PQS-1664(+)_Mut-1_, but not rvM-PQS-1664(+)_WT_, decreased the EC_50_ of CEP and PDS by a factor of ~500 and ~10, respectively (Fig 6C and 6D). While Ribavirin showed similar antiviral activity against rvM-PQS-1664(+)_WT_ and rvM-PQS-1664(+)_Mut-1_ (Fig 6E). These results indicated that CEP and PDS, but not Ribavirin, inhibit CCHFV infection in G4-dependent manners. Worth to note, rvM-PQS-1664(+)_Mut-1_ displayed greater resistance to CEP than PDS (Fig 6C and 6D). The most probable reason was that PDS is a broad-spectrum G4-binding ligand targeting both viral and host G4 structures, and the potential PDS host G4 targets may also play an antiviral role against CCHFV.

**Fig 7:**
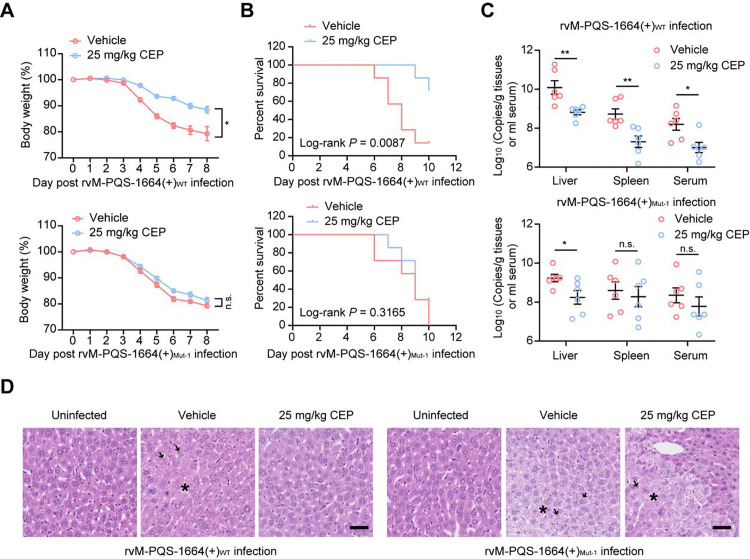
CEP protects mice from CCHFV pathogenesis by targeting G4. (A and B) Protective effects of CEP (25 mg/kg) on fatality caused by rvM-PQS-1664(+)_WT_ or rvM-PQS-1664(+)_Mut-1_ infection. Body weight (A) and survival (B) of the mice (n = 7) were monitored daily. (C and D) Protective effects of CEP (25 mg/kg) on rvM-PQS-1664(+)_WT_ or rvM-PQS-1664(+)_Mut-1_ caused pathogenesis. Viral cloads were measured using qRT-PCR and expressed as the number of viral RNA copies per microgram of tissue or per mL of serum (C). Hematoxylin and eosin (H&E) staining was conducted to demonstrate the pathological alterations. (D). The extensive necrosis (black asterisks) and necrotic cellular debris (black arrows) in liver were indicated. Scale bars: 100 μm. n.s., no significance. ^*^P < 0.05, ^**^P < 0.01 by Student’s *t* tes*t*.

We next evaluated whether CEP would impair CCHFV infection in C57BL/6 mouse model. Since C57BL/6 mice are naturally resistant to CCHF, we used anti-IFN type I receptor antibody to render C57BL/6 mice susceptible to CCHFV infection as reported previously [18]. The mice were inoculated with rvM-PQS-1664(+)_WT_ or rvM-PQS-1664(+)_Mut-1_ (100 TCID_50_ virus/mouse) via subcutaneous route, and treated with vehicle (control) and CEP (25 mg/kg intraperitoneally starting at 24 hours prior to virus inoculation and continuing until 5 days post infection). We found that CEP treatment significantly reduces body weight loss and lethality in the mice infected with rvM-PQS-1664(+)_WT_ (Fig 7A and 7B). In addition, the viral loads and pathological injury were dramatically reduced in the liver and spleen of mice treated with CEP (Fig 7C and 7D). And M-PQS-1664(+) G4-disruptive mutation results in the loss of the CEP target, thus the inhibition effects of CEP on rvM-PQS-1664(+)_Mut-1_ infection were less pronounced compared to those of CCHFV-G4_WT_, leading to a higher mortality rate in the CEP-treated mouse groups. These results suggested that CEP inhibits CCHFV infection by targeting M-PQS-1664(+) G4.

## Discussion

Despite significant advancements in comprehending the infection, immunity, and pathogenesis of CCHFV over recent decades, the establishment of effective strategies for controlling this virus remains elusive. Further exploration is warranted to identify novel avenues for CCHF treatment. Here, we have identified a G-rich RNA sequence within the CCHFV genome that has the ability to form a stable G4 structure. By performing *in vitro* EGFP and luciferase reporter gene assays, we found that M-PQS-1664(+) G4 formation can inhibit mRNA translation processes. Furthermore, our experimental results confirmed that CEP can bind and stabilize the M-PQS-1664(+) G4, exhibiting remarkable anti-CCHFV activity without any observable toxicity. Notably, the antiviral potency of CEP surpasses that of Ribavirin. Additionally, we had demonstrated that DDX60 has the ability to unfold the M-PQS-1664(+) G4 *in vitro* through fluorescence turn-on assays, and can enhances CCHFV infection. Collectively, these findings suggested that targeting M-PQS-1664(+) G4 holds promise for developing effective strategy to combat CCHFV.

In recent times, G4 structures have been detected in various organisms [43], including numerous viruses, and their role in controlling the viral life cycle has been established [21,32]. In the HIV genome, G4s found in the long terminal promoter (LTR) region serve as essential regulatory elements for viral transcription that are conserved over time [44–46]. The presence of G4s in SARS-CoV-2 has been observed to suppress nucleocapsid phosphoprotein expression, thereby hindering viral replication [24,26]. As a result, targeting G4 structures has emerged as a potential approach for antiviral strategies. However, the presence of G4 structures in the genome of CCHFV has not been systematically investigated. In this study, we identified several PQSs on both positive- and negative-sense strands of CCHFV and confirmed that M-PQS-1664(+) can form a stable G4 structure. Furthermore, our findings demonstrated that compounds such as PDS and CEP can stabilize M-PQS-1664(+) G4 to inhibit mRNA translation by performing EGFP and luciferase reporter gene assays. Antiviral assays indicated that CEP exhibits remarkable antiviral activity *in vitro* and *in vivo*. More importantly, CEP possessed antiviral activity (50% inhibitory concentration ~ 0.2 μM) that having ~ 88 × the potency of ribavirin. Conversely, we found that helicase DDX60 is capable of unfolding G4 structures *in vitro* by performing fluorescence turn-on assays. In addition, DDX60 was up-regulated during CCHFV infection and could promote CCHFV replication. These findings underscored the regulatory roles of G4s in the life cycle of CCHFV, thereby underscoring their significant potential as therapeutic targets against CCHF.

The enriched and conserved nature of viral G4s, along with their proposed roles in replication, transcription, translation and post-transcriptional, suggests that these elements are promising targets for inhibiting viral replication and infection. However, viral G4 formation is not necessarily detrimental to the virus. Epstein-Barr virus (EBV)-encoded nuclear antigen 1 (EBNA1) plays an essential role in viral genome maintenance but is also highly antigenic [47]. The formation of G4s within EBNA1 mRNA limits its expression to the minimal level required for functionality while simultaneously reducing immune recognition, thereby facilitating immune evasion by EBV [47]. A recent study characterized the formation of a conserved G4 structure within the polymerase-coding region of orthoflaviviruses in the *Flaviviridae* family, and viral G4s supports orthoflavivirus replication by stress reduction [48]. In this study, we found viral G4 in the mRNA of CCHFV glycoprotein inhibits glycoprotein expression to inhibit CCHFV replication. In addition, DDX60-mediated viral G4 unwinding and viral G4 mutations promote CCHFV replication. Similarly, a recent study showed that host DDX18 and viral nsp10, efficiently unwound the viral G4 structure, thereby facilitating porcine reproductive and respiratory syndrome virus (PRRSV) replication [49]. G4 ligands exhibit significant antiviral activity by stabilizing viral G4s. Viral evolution is driven by selective pressure, and if stabilizing G4 becomes disadvantageous, the virus may acquire mutations in these regions to evade treatment. Thus, in the course of treatment with G4-based drugs, viruses are likely to develop resistance to these compounds through mutations in the G4-forming regions, which represents one of the primary challenges confronting G4-targeted antiviral therapy. The potential for viral escape through mutations highlights the need for further research into mechanisms of resistance and the development of strategies to counteract such adaptations in the future.

The previous studies have demonstrated the crucial involvement of DDX60 in innate immunity against viral invasion [50,51]; however, it has also been reported that this helicase fails to detect viral nucleic acid and consequently does not initiate the IFN-I signaling cascade [52]. These perplexing findings necessitate further investigations to elucidate these ambiguous results. In our study, our results indicated that DDX60 is up-regulated in response to CCHFV infection, and then it is hijacked to unwind M-PQS-1664(+) G4 for facilitating viral replication. While CEP could stabilize the M-PQS-1664(+) G4 by blocking DDX60 mediated G4 unwinding, resulting in the inhibition of viral replication, suggesting that DDX60 may be a potential therapeutic target for CCHF. This finding would provide new insights into developing novel antiviral drugs against CCHFV.

Despite the promising potential of G4-based antiviral strategies, the lack of FDA-approved drugs specifically targeting G4 stabilization remains a significant hurdle. This primarily stems from the inadequate drug-like properties and selectivity profiles exhibited by conventional G4-specific ligands [32]. A recent study has performed a ligand-based pharmacophore virtual screening of FDA approved drugs to find candidates targeting viral RNA G4 structures [53]. Based on the FDA-approved drugs identified in the previous study, we conducted a screening of compounds that exhibit stabilizing effects on M-PQS-1664(+) G4. Here, we found that CEP shows excellent anti-CCHFV activity by targeting M-PQS-1664(+) G4. More important, CEP selectively stabilized M-PQS-1664(+) G4, but not host cellular DNA or RNA G4s. And our results from the animal experiments demonstrated the prophylactic efficacy of CEP against CCHFV infection. CEP, a naturally occurring bisbenzylisoquinoline alkaloid derived from the plant Stephania Cephalantha Hayata, stands as the sole approved bisbenzylisoquinoline alkaloid for human use and has been employed in clinical settings for over 70 years [54,55]. CEP can be delivered for extended durations at relatively high doses, through oral or intravenous routes, and it demonstrates safety and good tolerability, accompanied by minimal adverse effects [56]. In clinical practice, it has been utilized to manage snake bites, alopecia, malaria, and radiation-induced leukopenia [56]. CEP has been shown to regulate the activity of multiple cellular proteins, such as p53, caspase-9 and p38 etc [57]. For examples, CEP can inhibit NF-κB activation by blocking the IKK pathway [58]. CEP can inhibit the efflux function of P-glycoprotein [59]. Notably, existing research has demonstrated that CEP possesses significant antiviral bioactivity against a wide range of viruses. A recent study has demonstrated that CEP can inhibit SARS-CoV-2 spike protein/angiotensin-converting enzyme 2 (ACE2)-mediated membrane fusion [60]. Moreover, a phase I/II clinical trial application has been submitted to assess the efficacy of CEP in treating SARS-CoV-2 infections [61]. Consequently, the expedited approval process and reduced cost of clinical trials can be attributed to the availability of pre-existing absorption, distribution, metabolism, excretion, and toxicity data. Moreover, the presence of pertinent safety and pharmacology data for CEP significantly mitigates the risk of failure.

In summary, we have demonstrated the regulatory roles of G4s and DDX60 in the viral life cycle of CCHFV, highlighting their potential as the therapeutic targets for the development of more potent antiviral drug against CCHFV. Furthermore, CEP, an FDA-approved drug, holds promise for expedited clinical trials in treating CCHF. Combinatorial regimens involving agents from different classes that target multiple stages in the viral life cycle of CCHFV, such as the combination therapy comprising CEP and Ribavirin, may effectively prevent the emergence of drug-resistant mutants and represent a promising therapeutic approach for managing CCHFV infection.

## Materials and methods

### Ethics statement

CCHFV is classified as a BSL-3 agent in China, and handling CCHFV requires Biosafety Level 3 (BSL-3) or Animal Biosafety Level 3 (ABSL-3) facilities (https://view.officeapps.live.com/op/view.aspx?src=http%3A%2F%2Fwww.nhc.gov.cn%2Fqjjys%2Fs7948%2F202308%2Fb6b51d792d394fbea175e4c8094dc87e%2Ffiles%2F7b6b5be3d8bf4381ad5fb8d3cab2b5e7.docx&wdOrigin=BROWSELINK) [17,62,63]. All recombinant CCHFV (approval number IV220019) and animal experiments (approval number IACUC-DWCF-2023036) were approved by the Institutional Animal Care and Use Committee of Academy of Military Medical Sciences (approval number IACUC-DWCF-2023036). The *in vitro* antiviral studies were carried out in the BSL-3 facilities and the antiviral studies in animal models were carried out in the ABSL-3 facilities.

### Cells, oligonucleotides and compounds

The cell lines utilized in this study, including HEK293T, Huh7, HepG2, SW13, BSR-T7/5, and Vero E6, were sourced from the China Center for Type Culture Collection (CCTCC) located in Wuhan, China. These cell lines were maintained in high-glucose Dulbecco’s Modified Eagle’s Medium (DMEM) provided by HyClone (USA), enriched with 10% fetal bovine serum (FBS) from the same supplier, along with 100 units/mL of penicillin and 100 micrograms/mL of streptomycin. Cultures were incubated at 37 °C in a humidified atmosphere containing 5% CO_2_. Oligonucleotides required for the experiments were custom-synthesized by Shanghai Sangon Biological Engineering Technology & Services, based in Shanghai, China, with their specific sequences detailed in S2 Table. All chemical compounds employed in this research were procured from MedChemExpress (MCE) and used directly as received, without additional purification steps.

### Identification of CCHFV G4 sites and conservation analysis

The complete genetic sequences of CCHFV were obtained from the Genome database at the National Center for Biotechnology Information (NCBI) (https://www.ncbi.nlm.nih.gov/genomes). The FASTA sequences of the CCHFV genomes were then utilized to predict potential G4-forming sequences using Pqsfinder [64,65], QGRS-mapper [66], and G4Hunter [67,68]. For the Pqsfinder score, PQSs with a ratio exceeding an arbitrarily set threshold of 19 were deemed accessible under conditions that promote G4 folding. Regarding the QGRS-mapper score, PQSs were considered accessible under G4-favoring conditions if their ratio surpassed the arbitrary threshold of 17. For the G4Hunter score, PQSs were regarded as accessible under conditions conducive to G4 folding when their ratio was greater than the arbitrary cutoff of 1.5. The search was conducted based on the algorithm: G_≥2_N_1-15_G_≥2_N_1-15_G_≥2_N_1-15_G_≥2_, where G represents guanine and N represents any base including guanine.

To examine the preservation of CCHFV PQS sites among different strains, a total of 186 complete genomic sequences from the NCBI Genome database were collected. The aligned sequences were then visually presented using WebLogo 3 software [69] to create graphical representations.

### Fluorescence turn-on assays

For NMM and ThT treated samples, the concentration of RNA oligonucleotides, NMM and ThT was fixed at 2 μM. The 2 μM RNA oligonucleotides were incubated with 2 μM NMM or 2 μM ThT in 100 mM K^+^ buffer, respectively, and the mixtures were allowed to stand for 3 hours. For G4-unfolding by DDX60, The 2 μM RNA oligonucleotides were incubated with 2 μM NMM and the indicated concentration of DDX60 proteins (P05362; Solarbio, China) in presence of 1 mM ATP, and the mixtures were allowed to stand for 3 hours. The spectrofluorometer used for the assays was a JASCO FP-6500, and all measurements were conducted at room temperature. Emission spectra were obtained with an excitation wavelength of 399 nm for NMM-treated samples and 442 nm for ThT-treated samples.

### Circular dichroism (CD) measurements

The JASCO J-1500 spectropolarimeter equipped with a temperature-controlled water bath was employed to perform CD spectra and CD melting experiments. The 3 μM RNA oligonucleotides were incubated without or with 2 μM compounds in 100 mM K^+^ buffer, and the mixtures were allowed to stand in 4 °C overnight. Prior to usage, the optical chamber of the CD spectrometer was deoxygenated using dry purified nitrogen and maintained under a continuous nitrogen atmosphere throughout the experiments. During the CD melting experiments, measurements were recorded at a heating rate of 1 °C per minute in response to temperature variations.

### Polyacrylamide gel electrophoresis (PAGE)

The FAM-labeled RNA oligonucleotides were prepared in a 100 mM K^+^ buffer and subjected to thermal denaturation at 95 °C for 5 minutes, followed by gradual cooling to room temperature. Native gel electrophoresis was performed on a 20% acrylamide gel at 4 °C using 1 × TBE buffer containing 10 mM K^+^. Approximately 1 μM of RNAs was loaded onto the gel.

### MicroScale thermophoresis assays

The binding constant of the candidate compounds and proteins with CCHFV G4 were detected by performing MicroScale Thermophoresis (MST) assays, done by Bio-lab (Wuhan, China).

### Vectors construction and transfection

For EGFP reporter vectors constructs, pLV-EGFP-N vector (Inovogen Tech. Co., Beijing, China) were used as the template for PCR and engineered to harbor M-PQS-1664(+) and its mutant sequences sequence at the N-terminus of the EGFP reporter gene. The forward primer is designed to possess: a pendant 5’-segment comprising a NotI cleavage site and M-PQS-1664(+) or its mutant sequences, along with the 3’-region matching the N terminus of EGFP in frame. The reverse primer is designed to match the C terminus of EGFP. The PCR-amplified sequences were inserted into NotI and BsrGI digested pLV-EGFP-N vectors. The primers were listed in S2 Table. For luciferase reporter gene vectors constructs, we utilized pGL3-promoter vector. The M-PQS-1664(+) and its mutant sequences were synthesized by Shanghai Sangon Biological Engineering Technology & Services (Shanghai, China). The pGL3-promoter vectors harboring M-PQS-1664(+) sequences upstream of luciferase reporter gene (pGL3-M-PQS-1664(+)_WT_) were generated by inserting the M-PQS-1664(+) sequences into HindIII and NcoI digested pGL3-promoter vectors. The pGL3-promoter vectors harboring M-PQS-1664(+) mutant sequences upstream of luciferase reporter gene (pGL3-M-PQS-1664(+)_Mut_) were generated by inserting the M-PQS-1664(+) mutant sequences into HindIII and NcoI digested pGL3-promoter vectors. The NotI (#R3189S), BsrGI (#R3575S), HindIII (#R3104S) and NcoI (#R3193S) were purchased from New England BioLabs (CA, USA). For pLV-EGFP-N vector, the viral packaging and infection procedures followed previously established protocols [70]. In brief, HEK293T cells were transfected using Lipofectamine 2000 (Invitrogen, USA) according to the manufacturer’s protocol, along with the pLV-EGFP-N plasmid and viral packaging plasmids. Forty-eight hours post-transfection, the viral supernatant was harvested, passed through a 0.45 μm filter, and applied to target cells at a 1:100 dilution for 8 hours in the presence of 80 μg/mL polybrene. Following infection, cells were dissociated using trypsin, counted, and re-seeded at low density in 60 mm dishes. Six hours later, selection agents were introduced. Puromycin (#P8230, Solarbio Life Sciences, China) selection was maintained for 7 days. For transfection experiments, Lipofectamine 2000 (Invitrogen, CA, USA) was utilized to introduce the specified vectors into cells as per the manufacturer’s instructions. Cells were collected 48–72 hours after transfection.

### EGFP expression assays

The pLV-EGFP-N containing M-PQS-1664(+)_WT_ or M-PQS-1664(+)_Mut_ stable expressed HEK293T cells were treated with PDS or CEP as indicated concentration for 48 hours. Following PDS and CEP treatment, the cells were rinsed twice with 1 × PBS, then stabilized using 4% paraformaldehyde for a period of 10 minutes. Next, they were made permeable by treating with 0.5% Triton X-100 in PBS for 15 minutes. This was followed by a blocking step, where the cells were incubated for 30 minutes in a solution containing 3% BSA and 0.2% Triton X-100 in PBS. Prior to imaging, DAPI staining was performed for 2 minutes in the dark. Images were subsequently captured using a confocal laser-scanning microscope LSM 700 (Carl Zeiss, Germany).

### Luciferase assay

HEK293T cells were seeded at a density of 6 × 10^4^ cells per well in 24-well plates. One day after seeding, the cells were transfected with luciferase reporter constructs pGL3-M-PQS-1664(+)_WT_ or pGL3-M-PQS-1664(+)_Mut_ as well as pRL-CMV Renilla luciferase report vectors using Lipofectamine 2000. One day after transfecting, PDS or CEP as indicated concentration were directly added to the wells. Twenty-four hours following PDS or CEP treatment, luciferase activities were assessed using the Dual-Luciferase Reporter Assay Systems (Promega). The findings from these assays are presented as the percentage of Firefly luciferase activity relative to Renilla luciferase activity after normalization.

### Construction of recombinant CCHFV

Recombinant infectious CCHFV was produced using a reverse genetics system that has been previously described [14,71]. In brief, Huh7 cells were transfected with T7 promoter-based plasmids containing the complete anti-genomic sense CCHFV L, M, and S genome segments (strain IbAr10200) along with Pol II-expression plasmids providing CCHFV polymerase (RdRp), NP, and T7 polymerase. After 72 hours of transfection, the cell culture supernatants were collected and clarified through low-speed centrifugation before being used to infect BSR-T7/5 cells. The virus stocks obtained from BSR-T7/5 cells at 48 hours post infection were quantified using tissue culture infective dose 50 (TCID_50_) assays.

### Evaluation of antiviral activities of PDS and CEP

To evaluate the antiviral efficacy of PDS and CEP, Huh7, SW13 and Vero E6 cells were seeded at either 1 × 10^4^ cells/well (96-well plate) 16 hours prior to treatment with the indicated compounds. Cells were pre-treated with the different doses of the indicated compounds for 1 hour, and the virus was subsequently added to allow infection for 2 hours. The compounds were withdrawn when cells were infected with the virus, and subsequently reintroduced following the infection. At 72 hours post infection, ZsG fluorescence was determined. To determine viral titers, cells were plated in 96-well plates at a concentration of 1 × 10^4^ cells per well. The following day, cells were exposed to serial dilutions of the virus (ranging from 10^-1^ ~ 10^-12^) for one hour at 37 °C. Unabsorbed viral particles were removed by washing the wells three times with PBS. Subsequently, each well received 200 μL of maintenance medium (DMEM supplemented with 2% FBS), and the cultures were incubated for an additional 3–5 days. Cells exhibiting characteristic cytopathic effects were monitored daily, and the TCID_50_ was calculated using the Reed-Muench method.

### Cell viability assays

Briefly, HEK293T (5 × 10^4^ cells per well), SW13 (4 × 10^4^ cells per well), Huh7 (4 × 10^4^ cells per well) and HepG4 cells (4 × 10^4^ cells per well) were incubated with different concentrations of the compounds in 96-well plates for 48 hours, followed by measuring the cell viability by using Cell Counting Kit-8 (CCK-8) (Dojindo, Japan) according to the manufacturer’s instructions.

### RNA G4 pull-down assays

Briefly, the biotin-labeled M-PQS-1664(+) G4 sequences were folded into RNA G4 structure, and folded M-PQS-1664(+) G4 and control oligonucleotides were immobilized on streptavidin beads and used as baits for affinity enrichments of proteins from Huh7 cell cytosolic extracts. Next, proteins bound to the M-PQS-1664(+) G4 oligonucleotides and control samples (M-PQS-1664(+)_Mut_ and empty beads) were subjected to on-bead tryptic digestion followed by mass spectrometry analysis. Each RNA pull-down assay was performed independently for two times.

### Quantitative real-time polymerase chain reaction (qRT-PCR) assays

Total RNAs were extracted from cultured cells using RNApure tissue&cell kit according to the manufacturer’s protocol. The cDNAs were synthesized from 500 ng of total RNAs using PrimeScript RT Master Mix (RR036A; Takara, Japan) according to the manufacturer’s protocol. The qRT-PCR assays were performed using SYBR FAST qPCR kit (Kapa Biosystems, USA) at the BioRad IQ5 real-time PCR system (BioRad, Hercules, CA, USA). The relative expression levels of RNAs were calculated using the comparative C_t_ method. Melting curve analyses were performed on all PCRs to rule out the non-specific amplification.

### Western blotting assays

To analyze the protein content of whole-cell lysates, cells were lysed using RIPA buffer obtained from CWBIO in Beijing, China. The total proteins (20 μg) were then suspended in Laemmli buffer containing 63 mM Tris-HCl, 10% glycerol, 2% SDS, and 0.0025% bromophenol blue at pH 6.8. Subsequently, electrophoresis was performed on SDS-polyacrylamide gels to separate the proteins. Following this step, the proteins were transferred onto a polyvinylidene difluoride membrane. For detection purposes, specific antibodies against DDX60 (ab139807; Abcam, USA), viral glycoprotein (ADI-36121; # CABT-NS1040) or GAPDH (ab8245; Abcam, USA) were incubated with the membranes. To visualize the target proteins, anti-mouse or anti-rabbit secondary antibodies conjugated with horseradish peroxidase (HRP) were used for subsequent incubation steps. The immunoreactive bands were detected using a chemiluminescent substrate kit called SuperSignal West Pico obtained from Thermo Fisher Scientific based out of Waltham, MA, USA along with a Western blotting detection system provided by BioRad located in Hercules CA. In order to ensure accurate quantification and normalization of protein levels during western blotting assays, GAPDH was utilized as a loading control throughout these experiments.

### Electrophoretic mobility shift assay (EMSA)

For protein-G4 binding, 200 nM FAM-labeled RNA was incubated with full-length DDX60 protein (P05362; Solarbio, China) in a binding buffer containing 10mM Tris-HCl (pH7.5), 100 mM KCl, 10 μM ZnCl, 1 mM DTT and 5% glycerol on ice for 90 min. The samples were then loaded onto a 5% polyacrylamide gel in TBE buffer at 4 °C. The samples were run at 60 V at 4 °C for 90 min. The gels were imaged with Tanon-4600SF (Tanon, shanghai, China).

### CRISPR/Cas9 gene editing for DDX60 knockout cells

Genome editing using the CRISPR/Cas9 system was conducted with the pX330-U6-Chimeric_BB-CBh-hSpCas9 plasmid. Short guide RNAs (sgRNAs) targeting DDX60 were designed via the http://crispr.dbcls.jp/ [72], and their sequences are provided in S2Table. The sgRNAs were annealed by incubating them in NEBuffer 2 at 95 °C for 15 minutes, followed by gradual cooling to room temperature over a period of 5 hours. Annealed sgRNA pairs, diluted to a concentration of 0.1 pmol/µl, were inserted into the pX330-U6-Chimeric_BB-CBh-hSpCas9 plasmid through restriction-ligation cycles utilizing BbsI (#R3539S; New England BioLabs, CA, USA)and T4 DNA Ligases (#M0202S; New England BioLabs, CA, USA). Cells that were transfected with the recombinant plasmids for 24 hours were then plated into 96-well plates. Approximately 14 days later, surviving single-cell clones were selected, resuspended, and transferred to 12-well plates. These single clones were expanded and subsequently analyzed for the absence of DDX60 protein expression via western blot analysis using anti-DDX60 antibody (ab139807; Abcam, USA).

### RNA immunoprecipitation (RIP) assays

RIP was performed using Magna RIP RNA-Binding Protein Immunoprecipitation Kit (Millipore, Bedford, MA, USA) according to the manufacturer’s instructions. Briefly, the lysates of the recombinant CCHFV-infected Huh7 cells were mixed with RIP buffer containing specific antibodies against DDX60 (ab139807; Abcam, USA), with IgG (#2947; Cell Signaling Technology, Danvers, MA, USA) serving as a negative control. Following an overnight incubation at 4 °C with magnetic beads, the beads were subsequently washed. The complexes formed by cell lysate proteins and antibodies were then subjected to treatment with a digestion buffer, which included 0.1% SDS and 0.5 mg/mL proteinase K, at a temperature of 55 °C for a duration of 30 minutes. The co-precipitated RNAs were extracted and detected by qRT-PCR assays using specific primers listed in S2 Table. Data represent the mean ± standard error of mean (SEM) of three independent experiments.

### Time-of-addition experiment

A time-of-drug-addition assays were performed to investigate which stage of the CCHFV life cycle it is that CEP (500 nM) and PDS (500 nM) interferes with. Huh7 cells infected with CCHFV at MOI 0.1 were treated with CEP (500 nM) and PDS (500 nM) at time points indicated followed by incubation at 37 °C in 5% CO2. At 72 hours post infection, ZsG fluorescence was determined at 24 hours post infection and the inhibition rates of infection were analyzed.

### *In vivo* anti-CCHFV studies of CEP

All animal experiments were conducted in full compliance with the guidelines established by the Chinese Regulations for Laboratory Animals and Laboratory Animal-Requirements of Environment and Housing Facilities. The adult mice lacking the type I interferon (IFN) receptor were highly susceptible to CCHFV infection, resulting in acute disease with uniformly fatal outcomes. To make the mice susceptible to recombinant CCHFV infection, each mouse received an intraperitoneal injection of 2.5 mg anti-IFN type I receptor antibody MAR1-5A3 (#BE0241, BioXCell) at the time of infection. The CCHFV infection animal experiments were conducted in accordance with protocols established in prior studies [17,18]. The eight-week-old female C57BL/6 mice were challenged with recombinant CCHFV (100 TCID_50_/mouse) via subcutaneous injection. And the mice were administrated with 25 mg/kg CEP or vehicle intraperitoneally (i.p.) 24 h prior to challenge. 25 mg/kg CEP was given i.p. once daily for 5 days post infection (n = 7). All mice were evaluated daily for changes in body weight. Animals were humanely euthanized if they experienced a weight loss exceeding 20%, displayed difficulty in movement, or showed no reaction to tactile stimulation.

Blood, liver, and spleen samples were collected. Part of each sample was preserved in Trizol for RT-qPCR analysis, while the remaining portion was fixed in 4% paraformaldehyde for subsequent histopathological examination. After the experiment is completed, the mice body were placed in the designated freezer at the experimental animal center for storage, and classified and processed by the experimental animal center.

### Statistical analyses

All statistical analyses were performed using SPSS 13.0 (developed in Chicago, IL, USA) and GraphPad Prism (from La Jolla, CA, USA). For comparisons of quantitative data between two groups, we applied Student’s t-test. The findings are expressed as the mean ± SEM (Standard Error of Mean), with each experiment was repeated at least three times. Statistical significance is denoted as follows: ^*^P < 0.05, ^**^P < 0.01, ^***^P < 0.001.

## Supporting information

S1 Fig Identification and selection of PQSs in CCHFV genome. (**A** and **B**) Chemical structures of G4 specific small molecule N-methyl mesoporphyrin IX (NMM) (A) and Thioflavin T (ThT) (B). (**C**) ThT fluorescence turn-on assays for CCHFV PQS candidates. (**D**) ThT fluorescence turn-on assays for CCHFV PQSs and their mutants.
(TIF)

S2 Fig Characterization of M-PQS-1664(+) G4 formation. (**A**) CD spectroscopy of M-PQS-1664(+)_WT_ and M-PQS-1664(+)_Mut_. CD, circular dichroism. (**B**) The formation of M-PQS-1664(+)_WT_ G4 detected by nondenaturing polyacrylamide gel electrophoresis experiments. (**C**) Plots of melting temperature (*T*_m_) versus concentration of M-PQS-1664(+)_WT_.
(TIF)

S3 Fig Chemical structures of G4-stabilizing candidates. (**A**) Chemical structures of traditional G4-specific ligands. (**B**) Chemical structures of potential G4-stabilizing FDA-approved drugs.
(TIF)

S4 Fig The formation of M-PQS-1664(+) G4 inhibits EGFP expression and luciferase activity. (**A**) Schematic representation of the construction strategy for pLV-EGFP-N vectors harboring M-PQS-1664(+)_WT_ or M-PQS-1664(+)_Mut_. (**B**) CEP treatment inhibits the EGFP expression in HEK293T cells transfected with pLV-EGFP-N vectors harboring M-PQS-1664(+)_WT_ in a dose-dependent manner. Representative confocal images were demonstrated. Scale bars: 10 μm. The relative fluorescent value of EGFP in transfected and CEP-treated HEK293T cells was measured after CEP treatment for 48 hours. (**C**) PDS treatment inhibits the EGFP expression in HEK293T cells transfected with pLV-EGFP-N vectors harboring M-PQS-1664(+)_WT_ in a dose-dependent manner. Representative confocal images were demonstrated. Scale bars: 10 μm. The relative fluorescent value of EGFP in transfected and PDS-treated HEK293T cells was measured after PDS treatment for 48 hours. (**D**) Schematic representation of the construction strategy for luciferase vectors harboring M-PQS-1664(+)_WT_ or M-PQS-1664(+)_Mut_. (**E** and **F**) The luciferase activity in HEK293T cells transfected with luciferase vectors harboring M-PQS-1664(+)_WT_ or M-PQS-1664(+)_Mut_ and treated with indicated CEP (E) or PDS (F) was performed by luciferase reporter assays. The mean of triplicate wells is represented by each point, with error bars indicating the SEM. The graphs presented here are representative of three independent experiments. ^**^P < 0.01, ^***^P < 0.001 by Student’s *t* test.
(TIF)

S5 Fig The effect of CEP and PDS treatment on recombinant CCHFV. (**A**) Schematic representation of the L, M, and S genome segments of CCHFV (Strain IbAr10200). The S segment of CCHFV was genetically modified by incorporating the ZsGreen1 (ZsG) protein coding sequence fused to the P2A sequence, positioned upstream of the NP coding region. The positions of M-PQS-1664(+) were annotated within the M segment. (**B**) Representative fluorescent microscopy images of CCHFV/ZsG-infected Huh7, SW13 and Vero E6 cells infected with CCHFV/ZsG at multiplicity of infection (MOI) of 0.1. (**C**) 500 nM CEP or 500 nM PDS treatment decreases the ZsG fluorescence. The cells were infected without or with CCHFV/ZsG at MOI 0.1., and the ZsG fluorescence (green) was determined at 72 hours post infection. The mean of triplicate wells is represented by each point, with error bars indicating the SEM. The graphs presented here are representative of three independent experiments. ^***^P < 0.001 by Student’s *t* test.
(TIF)

S6 Fig The cytotoxicity of CEP and PDS treatment in HEK293T, Huh7, SW13 and Vero E6 cells. (**A**) The cell viability of HEK293T, Huh7, SW13 and Vero E6 cells treated with CEP for 48 hours was quantitatively analyzed by performing CCK8 assays. (**B**) The cell viability of HEK293T, Huh7, SW13 and Vero E6 cells treated with PDS for 48 hours was quantitatively analyzed by performing CCK8 assays. The mean of triplicate wells is represented by each point, with error bars indicating the SEM. The graphs presented here are representative of three independent experiments.
(TIF)

S7 Fig The CCHFV infection induces DDX60 expression. (**A**) Volcano plot of differentially expressed genes in Huh7 cells relative to Huh7 cells infected with CCHFV. (**B**) Volcano plot of differentially expressed genes in HepG2 cells relative to HepG2 cells infected with CCHFV. (**C**) The protein levels of viral glycoprotein (Gc) in DDX60 WT (Ctrl) and DDX60 knockout (DDX60-KO) Huh7 cells as well as DDX60-KO Huh7 cells transiently transfecting DDX60. The cells were infected with CCHFV/ZsG at MOI 0.1., subsequently the protein levels of Gc were detected at 72 hours post infection by western blotting assays. (**D**) The cell viability of HepG2 cells transfected with si-scramble or si-DDX60 was quantitatively analyzed after transfection for 48 hours by performing CCK8 assays. (**E**) The cell viability of Huh7 cells transfected with si-scramble or si-DDX60 was quantitatively analyzed after transfection for 48 hours by performing CCK8 assays. The mean of triplicate wells is represented by each point, with error bars indicating the SEM. The graphs presented here are representative of three independent experiments.
(TIF)

S8 Fig PDS and CEP inhibited DDX60-mediated promotion of CCHFV replication. 500 nM CEP or 500 nM PDS treatment decrease the ZsG fluorescence in the recombinant CCHFV-infected DDX60-KO Huh7 cells without or with DDX60-OE. DDX60-KO, DDX60 knockout. DDX60-OE, DDX60 overexpression. The cells were infected with CCHFV/ZsG at MOI 0.1., and the ZsG fluorescence (green) was determined at 72 hours post infection. The mean of triplicate wells is represented by each point, with error bars indicating the SEM. The graphs presented here are representative of three independent experiments. ^**^P < 0.01, ^***^P < 0.001 by Student’s *t* test.
(TIF)

S9 Fig Time-of-addition experiment of CEP and PDS. (**A-C**) The scheme shows the experimental design and the period of cell-drug incubation (A). Huh7 cells were incubated with 500 nM CEP (B) or 500 nM PDS (C) at the time points indicated. The cells were infected with CCHFV/ZsG at MOI 0.1., and the ZsG fluorescence (green) was determined at 24 hours post infection and the inhibition rates of infection were analyzed. The mean of triplicate wells is represented by each point, with error bars indicating the SEM. The graphs presented here are representative of three independent experiments. n.s., no significance. ^***^P < 0.001 by Student’s *t* test.
(TIF)

S10 Fig PDS combined with ribavirin inhibits CCHFV infection. (**A**) The Huh7, SW13, and Vero E6 cells were treated with the indicated compounds either individually or in combination at the following concentrations: PDS (100 nM) and ribavirin (10 μM). Subsequently, the cells were infected with CCHFV at MOI 0.1, and the ZsG fluorescence (green) was determined at 72 hours post infection. Each data point represents the mean value obtained from triplicate wells, with error bars indicating standard deviation. (**B**) Cell viability was determined concurrently using the indicated compounds in Huh7, SW13, and Vero E6 cells. Each data point represents the mean value obtained from quadruplicate wells, with error bars indicating SEM.
(TIF)

S11 Fig Determination of G4-disruptive mutations. (**A**) NMM fluorescence turn-on assays for CCHFV G4-disruptive mutations. (**B**) ThT fluorescence turn-on assays for CCHFV G4-disruptive mutations. (**C**) The luciferase activity in HEK293T cells transfected with luciferase vectors harboring M-PQS-1664(+)_WT_ or M-PQS-1664(+)_Mut-1_ was detected after 500 nM CEP or 500 nM PDS treatment for 24 hours by performing luciferase reporter assays. The mean of triplicate wells is represented by each point, with error bars indicating the SEM. The graphs presented here are representative of three independent experiments.
(TIF)

S12 Fig The rvM-PQS-1664(+)_Mut_ exhibits a significantly reduced sensitivity to DDX60 knockout. (**A**) The protein levels of viral glycoprotein (Gc) in DDX60 knockout (DDX60-KO) Huh7 cells. The cells were infected with rvM-PQS-1664(+)_WT_ or rvM-PQS-1664(+)_Mut_ at MOI 0.1., subsequently the protein levels of Gc were detected at 72 hours post infection by western blotting assays. (**B**) DDX60 cannot interact with the CCHFV RNA with M-PQS-1664(+)_Mut-1_ in the rvM-PQS-1664(+)_Mut-1_ infected Huh7 cells, detected by performing RNA immunoprecipitation (RIP) assays. The cells were infected with rvM-PQS-1664(+)_WT_ or rvM-PQS-1664(+)_Mut-1_ at MOI 0.1., and the enrichment of CCHFV RNA containing M-PQS-1664(+)_WT_ or rvM-PQS-1664(+)_Mut-1_ by anti-DDX60 was determined at 48 hours post infection by performing RNA immunoprecipitation (RIP) assays. (**C** and **D**) The DDX60 WT (Ctrl) and DDX60 knockout (DDX60-KO) Huh7 (B) and HepG2 (C) cells as well as DDX60-KO Huh7 and HepG2 cells transiently transfecting DDX60 were infected without or with rvM-PQS-1664(+)_WT_ or rvM-PQS-1664(+)_Mut_ at MOI 0.1., subsequently the ZsG fluorescence (green) was determined at 72 hours post infection. The mean of triplicate wells is represented by each point, with error bars indicating the SEM. The graphs presented here are representative of three independent experiments. ^**^P < 0.01 by Student’s *t* test.
(TIF)

S1 Table Characteristics of the 25 putative G-quadruplexes in CCHFV genome. (XLSX)

S2 Table Sequence of the oligonucleotides used in this study. (XLSX)

S3 Table The strains and seuqences used for the analysis of conservation. (XLSX)

S4 Table Differentially expressed genes in HepG2 cells relative to HepG2 cells infected with CCHFV. (XLSX)

S5 Table Differentially expressed genes in Huh7 cells relative to Huh7 cells infected with CCHFV. (XLSX)

S6 Table Numerical source data. (XLSX)
