# Supplementary figures and images for: Viral hijacking of host DDX60 promotes Crimean-Congo haemorrhagic fever virus replication via G-quadruplex unwinding
Source: PLoS Pathog. 2025 Jun 27;21(6):e1013278. doi: 10.1371/journal.ppat.1013278 (PMC12221184; doi:10.1371/journal.ppat.1013278)

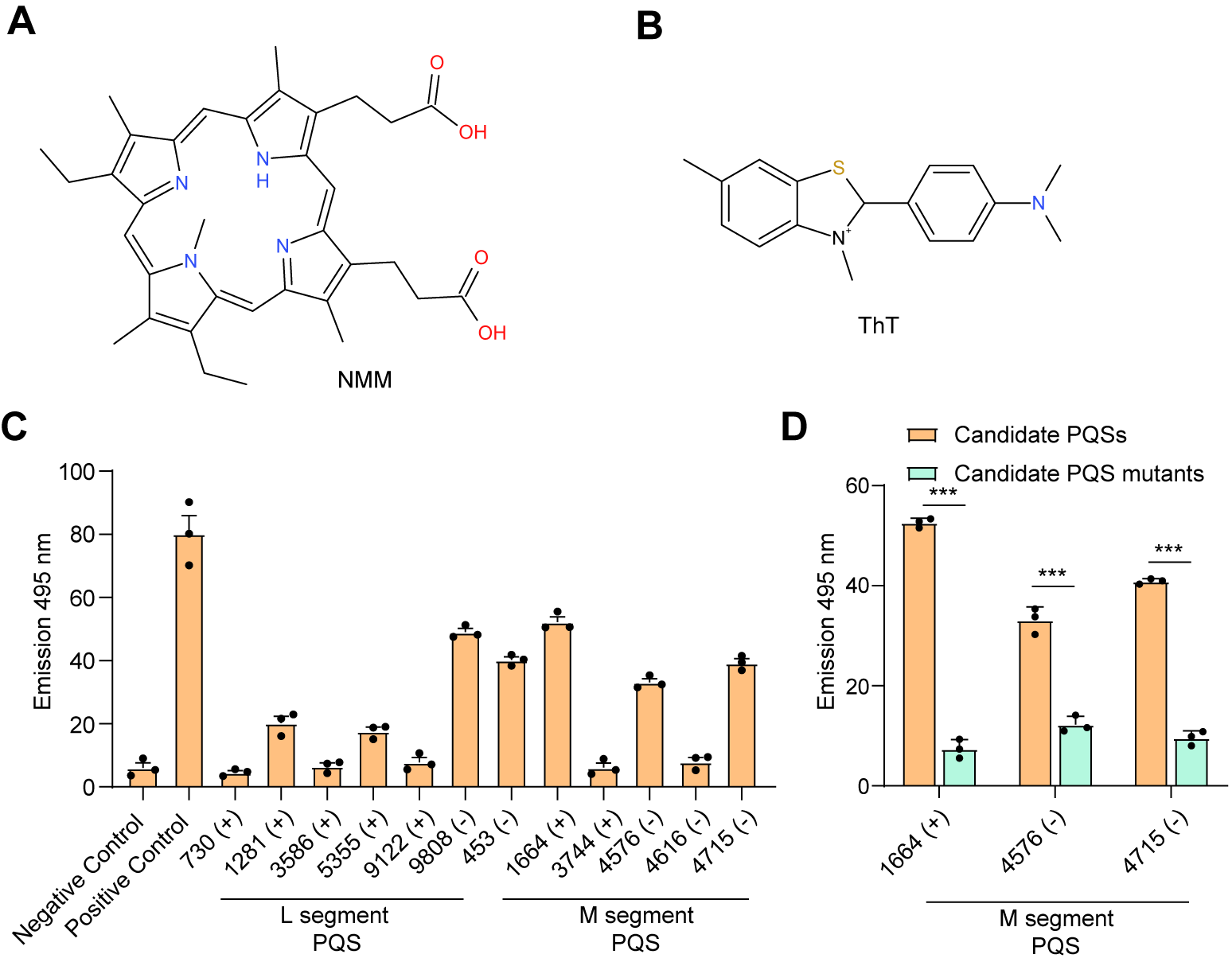

Supplement: S1 Fig — (A and B) Chemical structures of G4 specific small molecule N-methyl mesoporphyrin IX (NMM) (A) and Thioflavin T (ThT) (B). (C) ThT fluorescence turn-on assays for CCHFV PQS candidates. (D) ThT fluorescence turn-on assays for CCHFV PQSs and their mutants. (TIF) [file ppat.1013278.s001.tif]

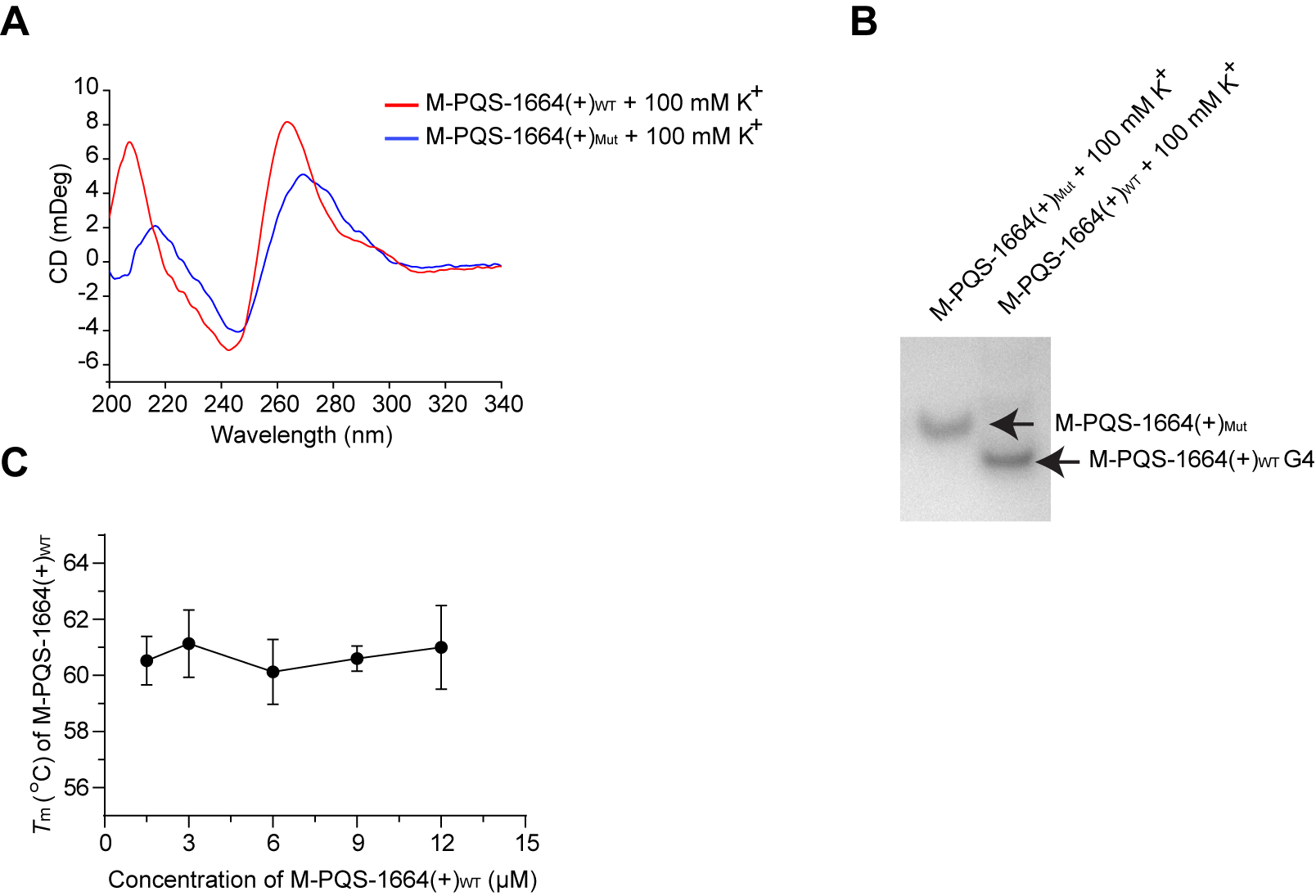

Supplement: S2 Fig — (A) CD spectroscopy of M-PQS-1664(+)WT and M-PQS-1664(+)Mut. CD, circular dichroism. (B) The formation of M-PQS-1664(+)WT G4 detected by nondenaturing polyacrylamide gel electrophoresis experiments. (C) Plots of melting temperature (Tm) versus concentration of M-PQS-1664(+)WT. (TIF) [file ppat.1013278.s002.tif]

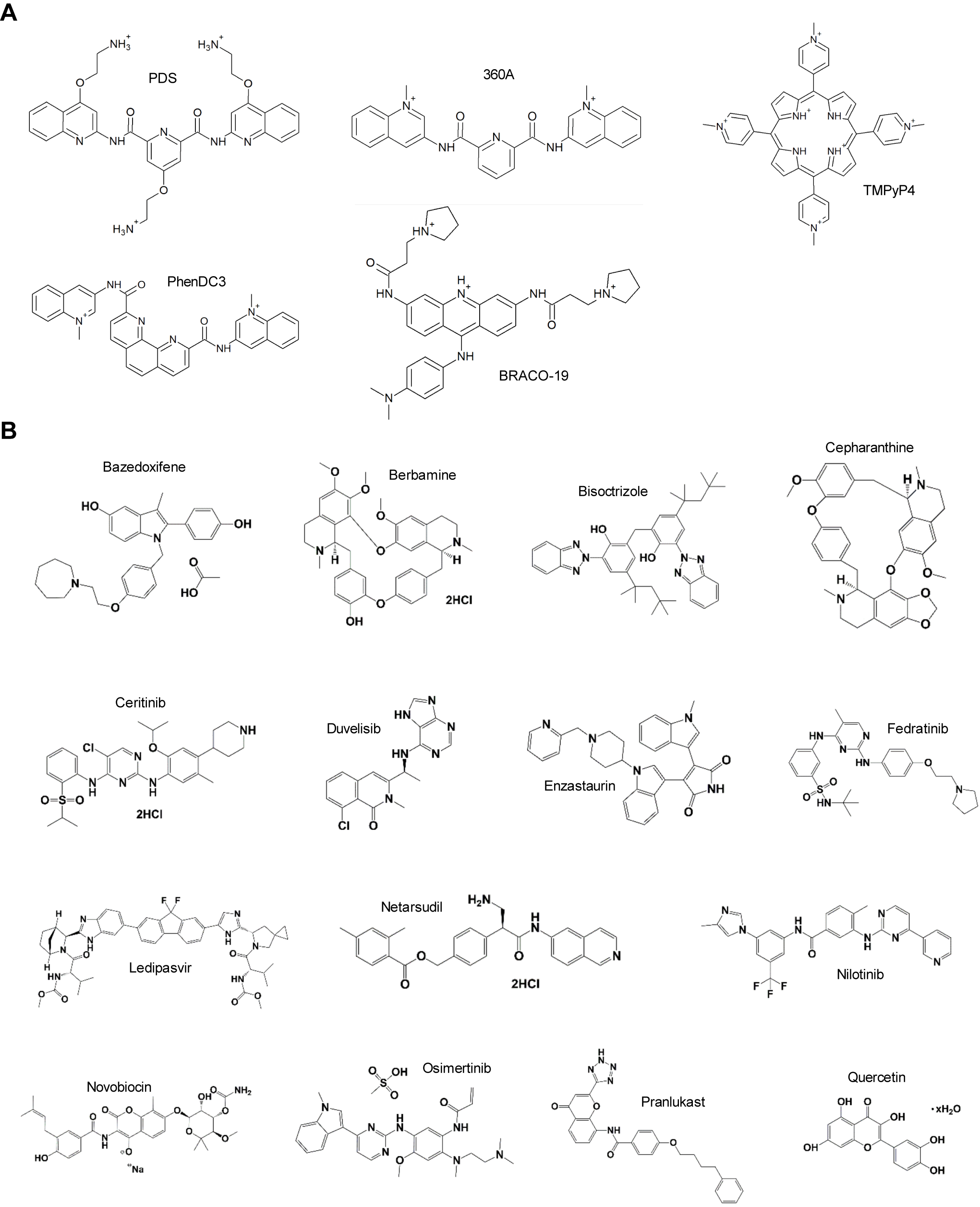

Supplement: S3 Fig — (A) Chemical structures of traditional G4-specific ligands. (B) Chemical structures of potential G4-stabilizing FDA-approved drugs. (TIF) [file ppat.1013278.s003.tif]

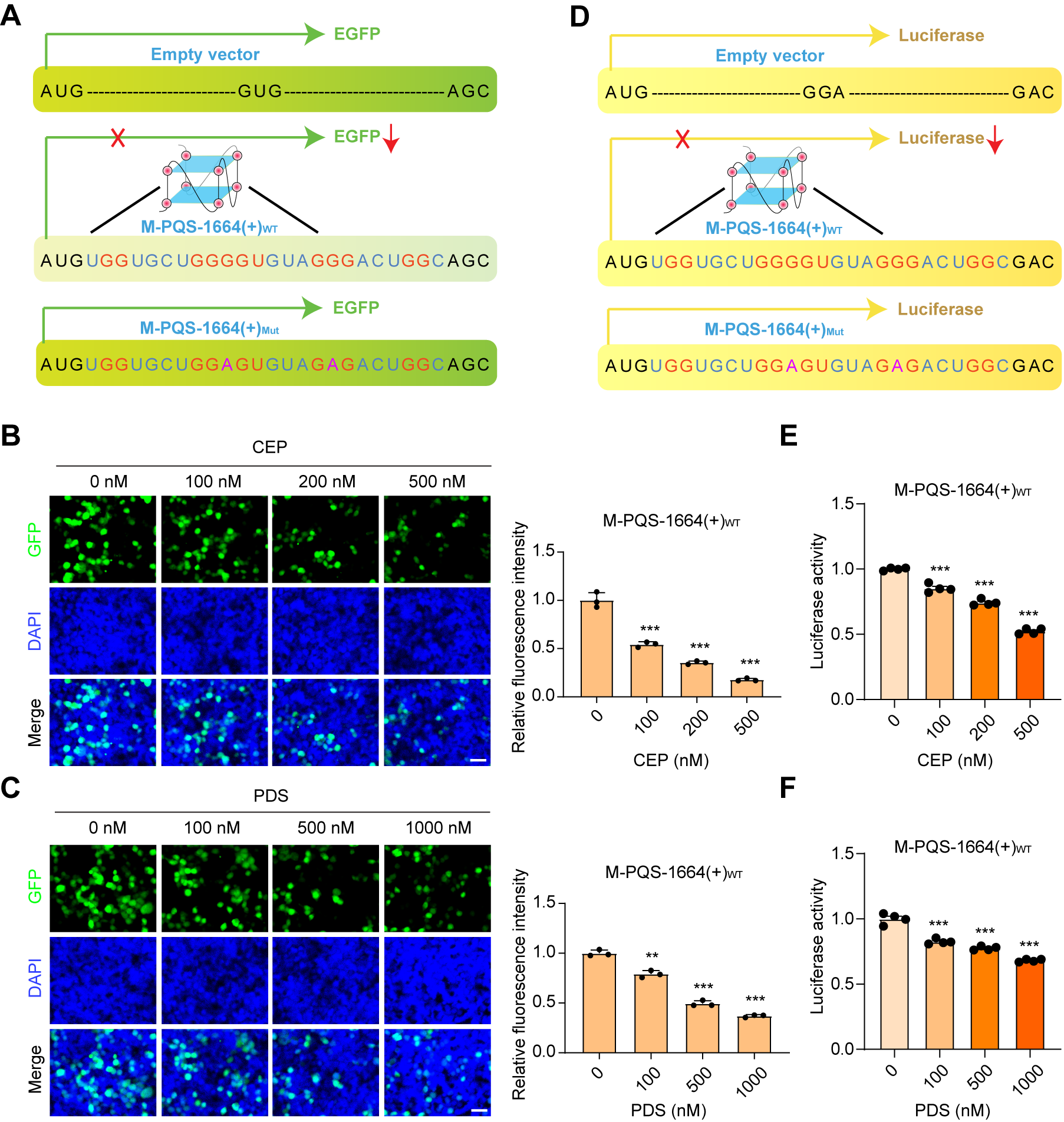

Supplement: S4 Fig — (A) Schematic representation of the construction strategy for pLV-EGFP-N vectors harboring M-PQS-1664(+)WT or M-PQS-1664(+)Mut. (B) CEP treatment inhibits the EGFP expression in HEK293T cells transfected with pLV-EGFP-N vectors harboring M-PQS-1664(+)WT in a dose-dependent manner. Representative confocal images were demonstrated. Scale bars: 10 μm. The relative fluorescent value of EGFP in transfected and CEP-treated HEK293T cells was measured after CEP treatment for 48 hours. (C) PDS treatment inhibits the EGFP expression in HEK293T cells transfected with pLV-EGFP-N vectors harboring M-PQS-1664(+)WT in a dose-dependent manner. Representative confocal images were demonstrated. Scale bars: 10 μm. The relative fluorescent value of EGFP in transfected and PDS-treated HEK293T cells was measured after PDS treatment for 48 hours. (D) Schematic representation of the construction strategy for luciferase vectors harboring M-PQS-1664(+)WT or M-PQS-1664(+)Mut. (E and F) The luciferase activity in HEK293T cells transfected with luciferase vectors harboring M-PQS-1664(+)WT or M-PQS-1664(+)Mut and treated with indicated CEP (E) or PDS (F) was performed by luciferase reporter assays. The mean of triplicate wells is represented by each point, with error bars indicating the SEM. The graphs presented here are representative of three independent experiments. **P < 0.01, ***P < 0.001 by Student’s t test. (TIF) [file ppat.1013278.s004.tif]

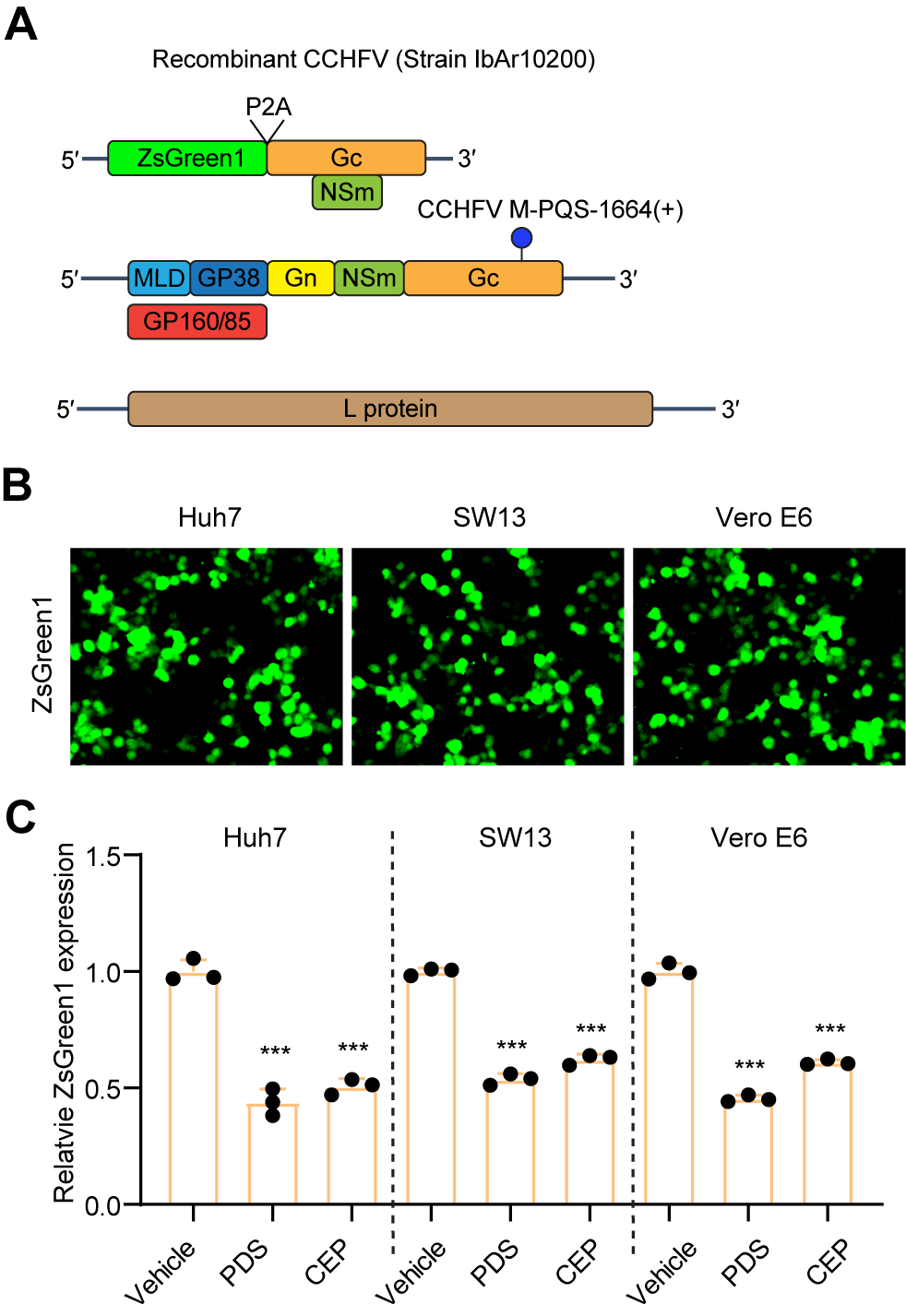

Supplement: S5 Fig — (A) Schematic representation of the L, M, and S genome segments of CCHFV (Strain IbAr10200). The S segment of CCHFV was genetically modified by incorporating the ZsGreen1 (ZsG) protein coding sequence fused to the P2A sequence, positioned upstream of the NP coding region. The positions of M-PQS-1664(+) were annotated within the M segment. (B) Representative fluorescent microscopy images of CCHFV/ZsG-infected Huh7, SW13 and Vero E6 cells infected with CCHFV/ZsG at multiplicity of infection (MOI) of 0.1. (C) 500 nM CEP or 500 nM PDS treatment decreases the ZsG fluorescence. The cells were infected without or with CCHFV/ZsG at MOI 0.1., and the ZsG fluorescence (green) was determined at 72 hours post infection. The mean of triplicate wells is represented by each point, with error bars indicating the SEM. The graphs presented here are representative of three independent experiments. ***P < 0.001 by Student’s t test. (TIF) [file ppat.1013278.s005.tif]

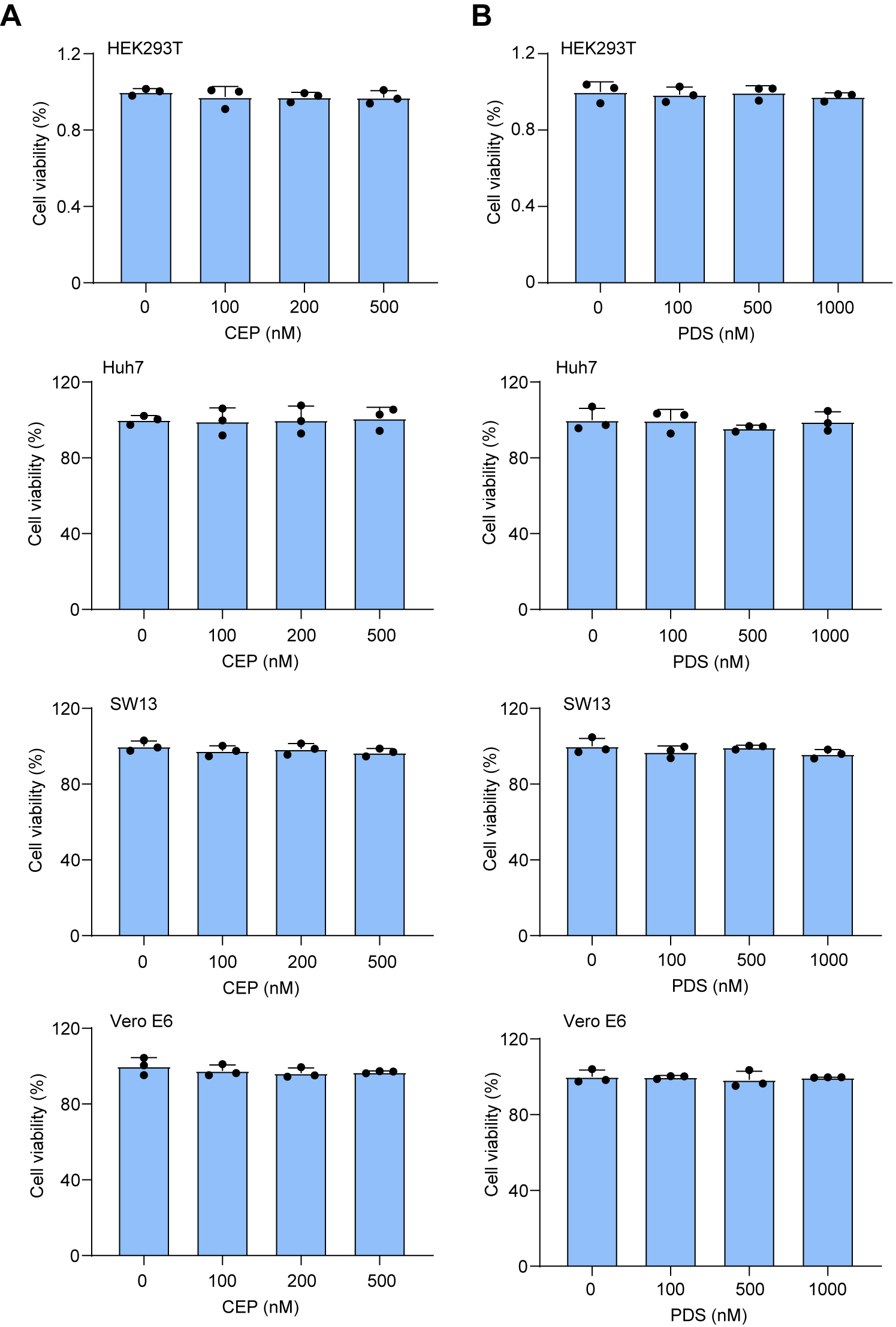

Supplement: S6 Fig — (A) The cell viability of HEK293T, Huh7, SW13 and Vero E6 cells treated with CEP for 48 hours was quantitatively analyzed by performing CCK8 assays. (B) The cell viability of HEK293T, Huh7, SW13 and Vero E6 cells treated with PDS for 48 hours was quantitatively analyzed by performing CCK8 assays. The mean of triplicate wells is represented by each point, with error bars indicating the SEM. The graphs presented here are representative of three independent experiments. (TIF) [file ppat.1013278.s006.tif]

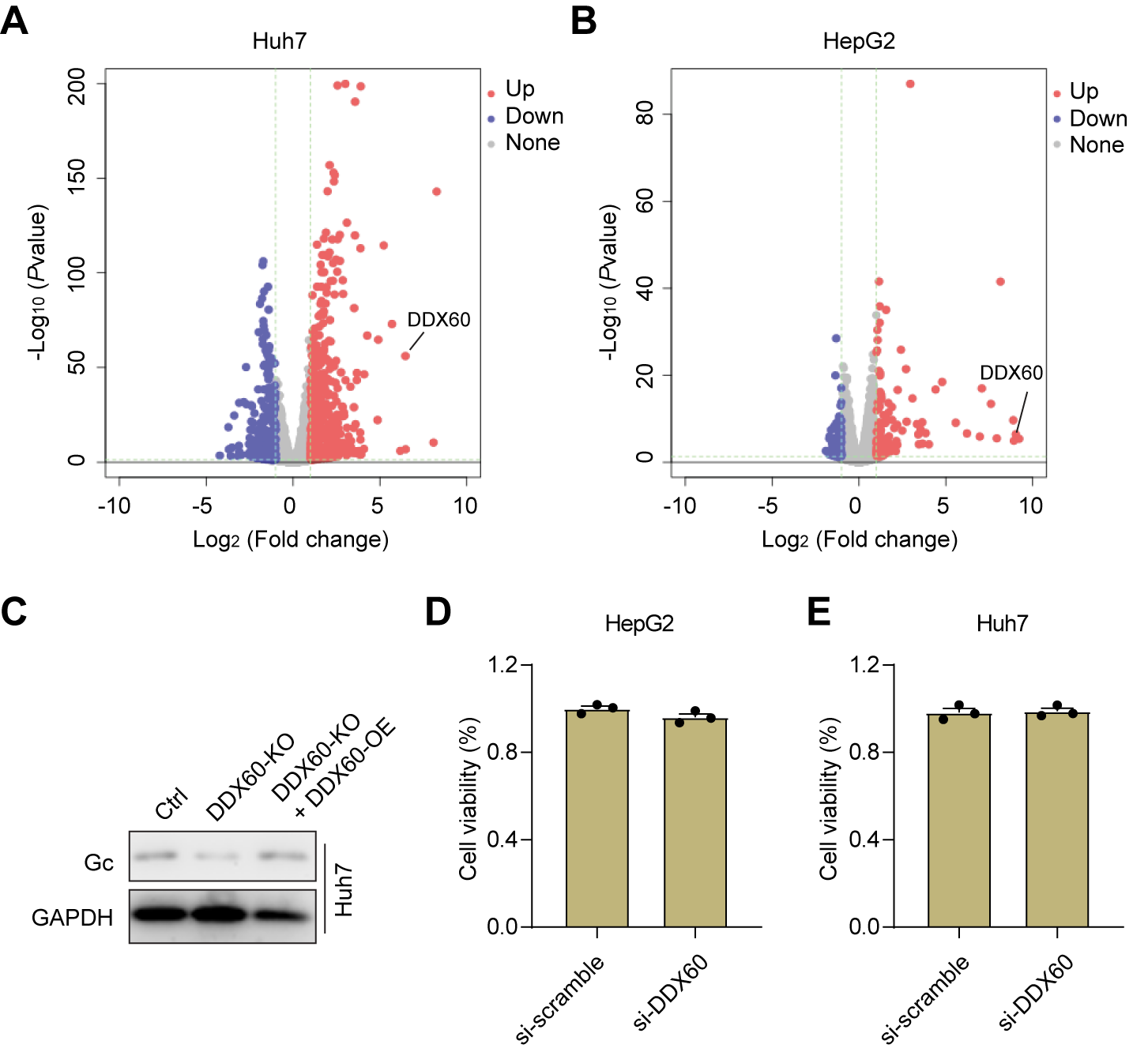

Supplement: S7 Fig — (A) Volcano plot of differentially expressed genes in Huh7 cells relative to Huh7 cells infected with CCHFV. (B) Volcano plot of differentially expressed genes in HepG2 cells relative to HepG2 cells infected with CCHFV. (C) The protein levels of viral glycoprotein (Gc) in DDX60 WT (Ctrl) and DDX60 knockout (DDX60-KO) Huh7 cells as well as DDX60-KO Huh7 cells transiently transfecting DDX60. The cells were infected with CCHFV/ZsG at MOI 0.1., subsequently the protein levels of Gc were detected at 72 hours post infection by western blotting assays. (D) The cell viability of HepG2 cells transfected with si-scramble or si-DDX60 was quantitatively analyzed after transfection for 48 hours by performing CCK8 assays. (E) The cell viability of Huh7 cells transfected with si-scramble or si-DDX60 was quantitatively analyzed after transfection for 48 hours by performing CCK8 assays. The mean of triplicate wells is represented by each point, with error bars indicating the SEM. The graphs presented here are representative of three independent experiments. (TIF) [file ppat.1013278.s007.tif]

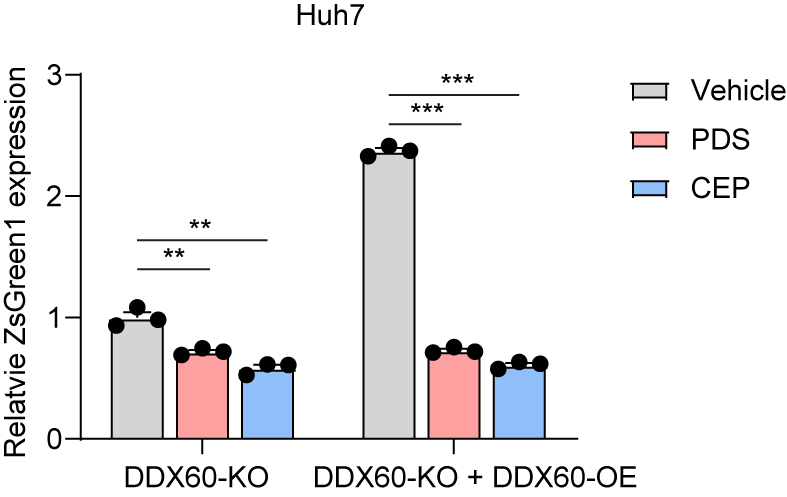

Supplement: S8 Fig — 500 nM CEP or 500 nM PDS treatment decrease the ZsG fluorescence in the recombinant CCHFV-infected DDX60-KO Huh7 cells without or with DDX60-OE. DDX60-KO, DDX60 knockout. DDX60-OE, DDX60 overexpression. The cells were infected with CCHFV/ZsG at MOI 0.1., and the ZsG fluorescence (green) was determined at 72 hours post infection. The mean of triplicate wells is represented by each point, with error bars indicating the SEM. The graphs presented here are representative of three independent experiments. **P < 0.01, ***P < 0.001 by Student’s t test. (TIF) [file ppat.1013278.s008.tif]

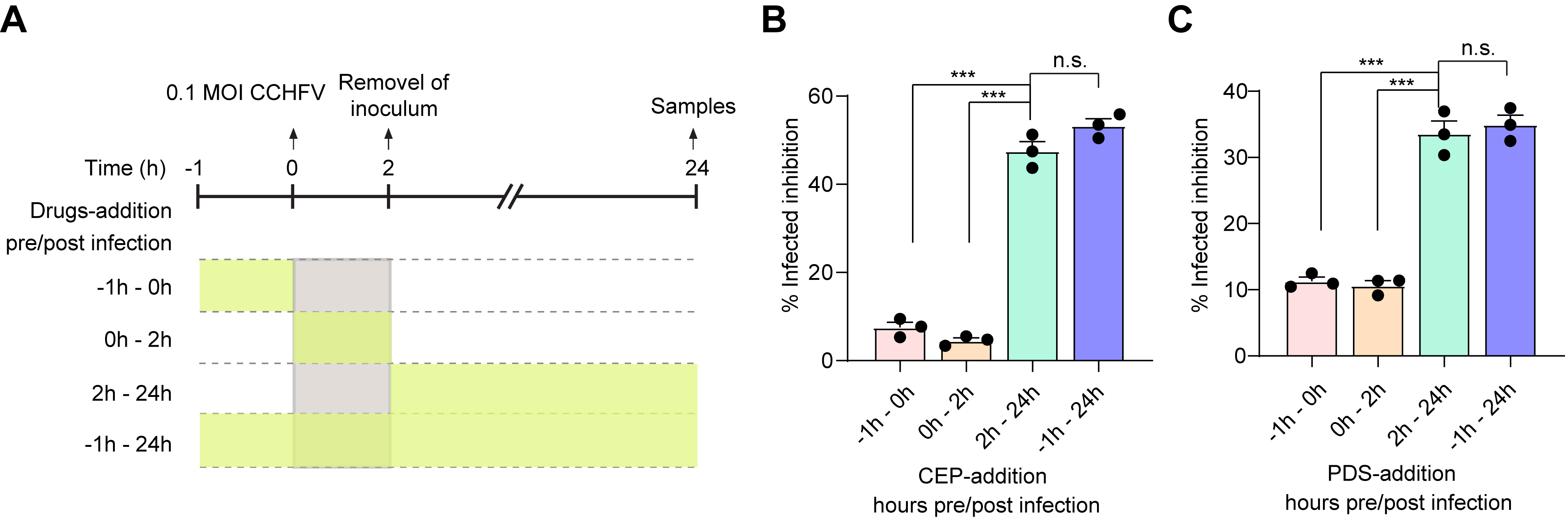

Supplement: S9 Fig — (A-C) The scheme shows the experimental design and the period of cell-drug incubation (A). Huh7 cells were incubated with 500 nM CEP (B) or 500 nM PDS (C) at the time points indicated. The cells were infected with CCHFV/ZsG at MOI 0.1., and the ZsG fluorescence (green) was determined at 24 hours post infection and the inhibition rates of infection were analyzed. The mean of triplicate wells is represented by each point, with error bars indicating the SEM. The graphs presented here are representative of three independent experiments. n.s., no significance. ***P < 0.001 by Student’s t test. (TIF) [file ppat.1013278.s009.tif]

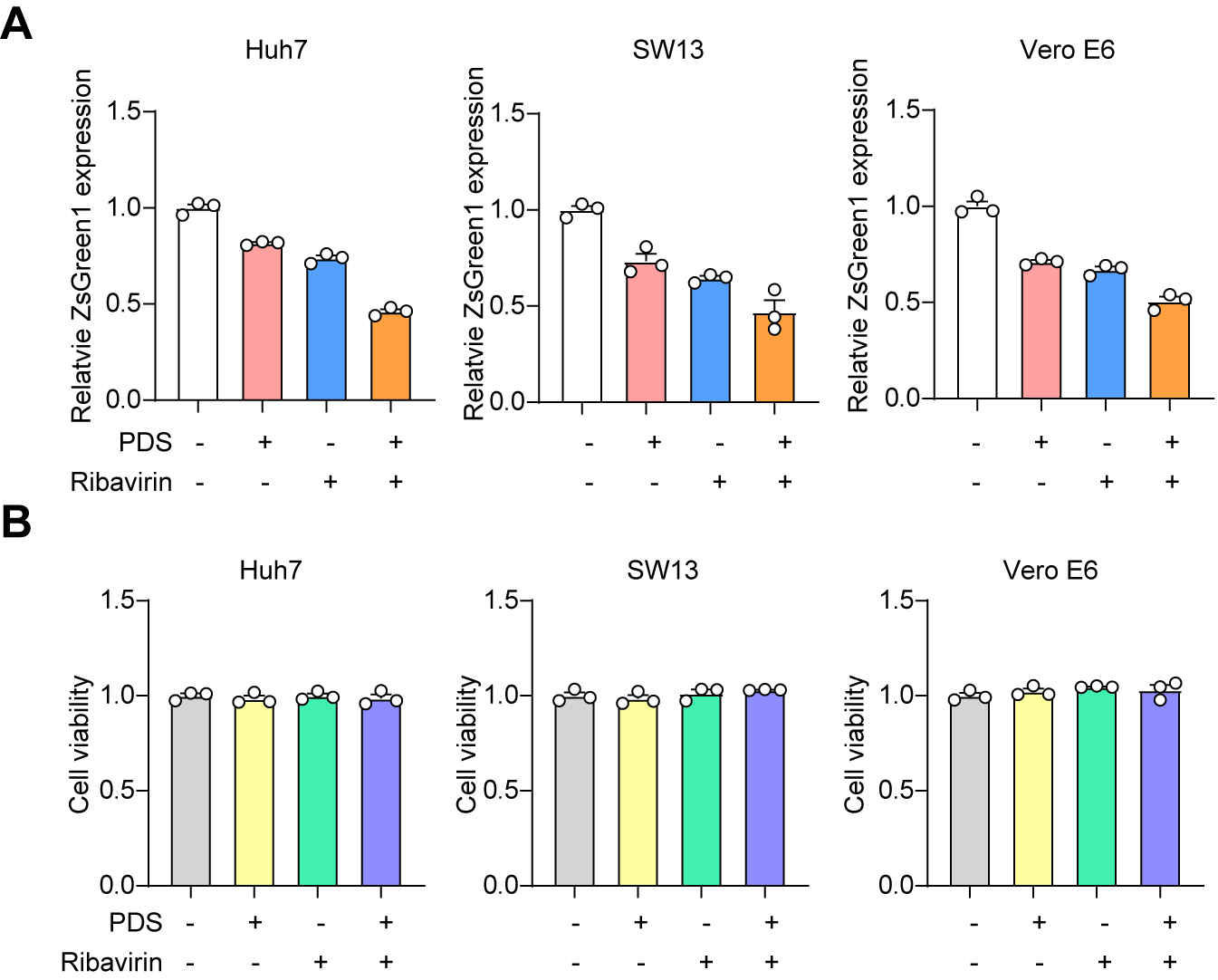

Supplement: S10 Fig — (A) The Huh7, SW13, and Vero E6 cells were treated with the indicated compounds either individually or in combination at the following concentrations: PDS (100 nM) and ribavirin (10 μM). Subsequently, the cells were infected with CCHFV at MOI 0.1, and the ZsG fluorescence (green) was determined at 72 hours post infection. Each data point represents the mean value obtained from triplicate wells, with error bars indicating standard deviation. (B) Cell viability was determined concurrently using the indicated compounds in Huh7, SW13, and Vero E6 cells. Each data point represents the mean value obtained from quadruplicate wells, with error bars indicating SEM. (TIF) [file ppat.1013278.s010.tif]

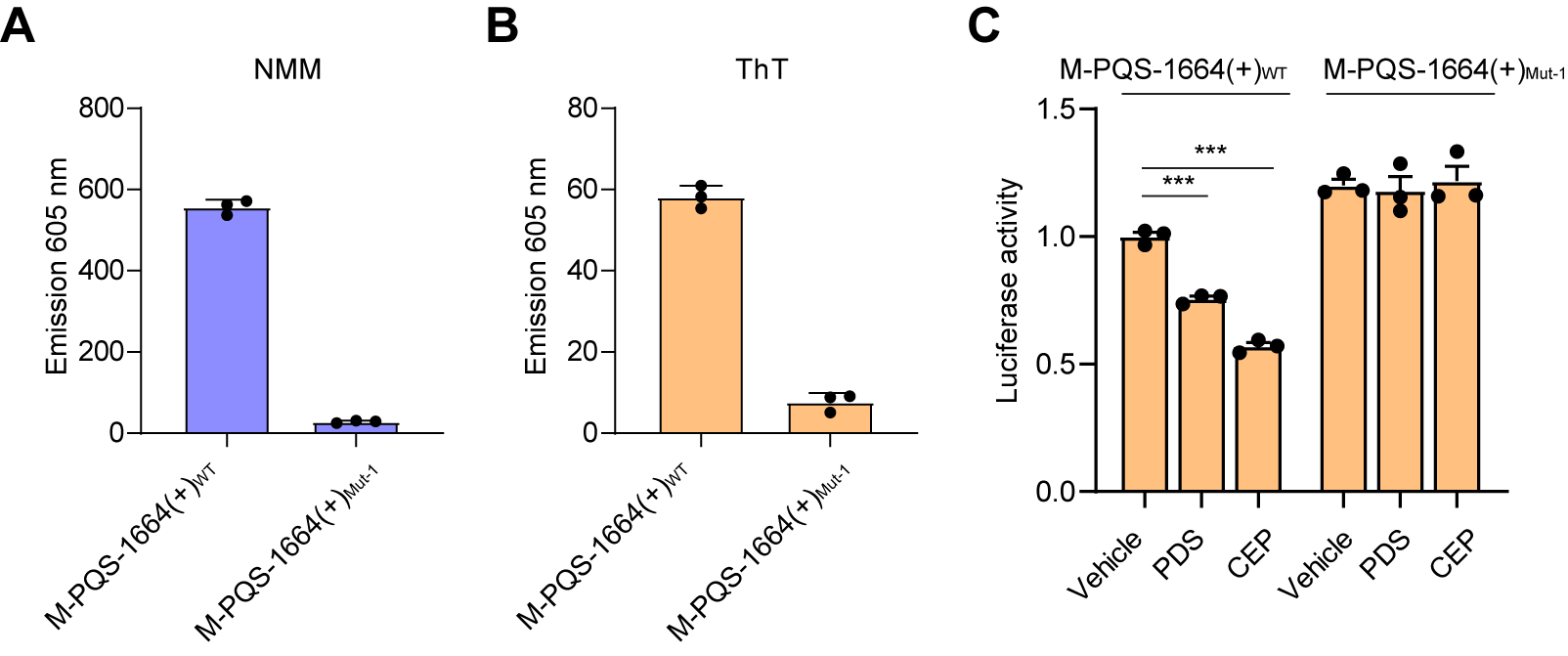

Supplement: S11 Fig — (A) NMM fluorescence turn-on assays for CCHFV G4-disruptive mutations. (B) ThT fluorescence turn-on assays for CCHFV G4-disruptive mutations. (C) The luciferase activity in HEK293T cells transfected with luciferase vectors harboring M-PQS-1664(+)WT or M-PQS-1664(+)Mut-1 was detected after 500 nM CEP or 500 nM PDS treatment for 24 hours by performing luciferase reporter assays. The mean of triplicate wells is represented by each point, with error bars indicating the SEM. The graphs presented here are representative of three independent experiments. (TIF) [file ppat.1013278.s011.tif]

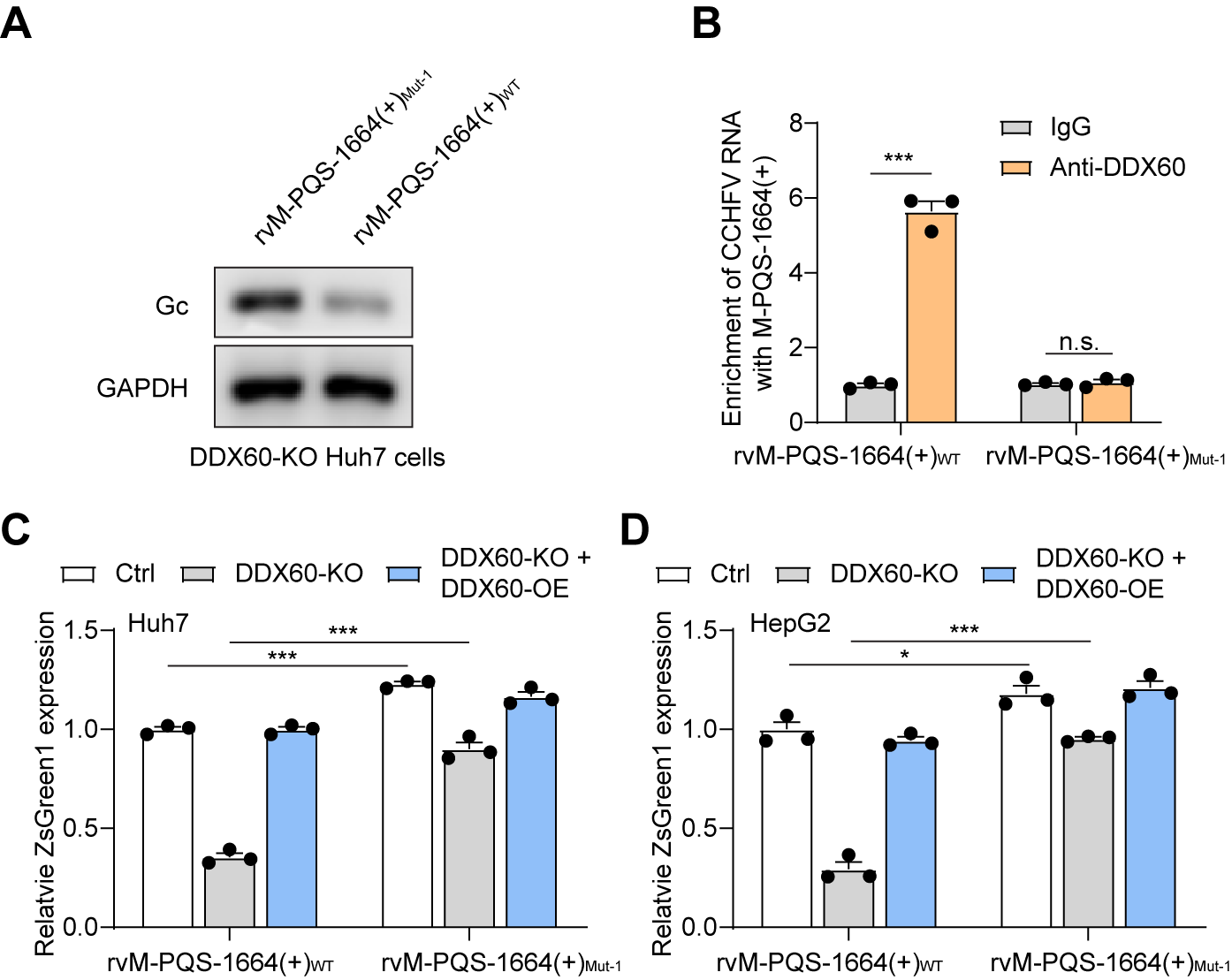

Supplement: S12 Fig — (A) The protein levels of viral glycoprotein (Gc) in DDX60 knockout (DDX60-KO) Huh7 cells. The cells were infected with rvM-PQS-1664(+)WT or rvM-PQS-1664(+)Mut at MOI 0.1., subsequently the protein levels of Gc were detected at 72 hours post infection by western blotting assays. (B) DDX60 cannot interact with the CCHFV RNA with M-PQS-1664(+)Mut-1 in the rvM-PQS-1664(+)Mut-1 infected Huh7 cells, detected by performing RNA immunoprecipitation (RIP) assays. The cells were infected with rvM-PQS-1664(+)WT or rvM-PQS-1664(+)Mut-1 at MOI 0.1., and the enrichment of CCHFV RNA containing M-PQS-1664(+)WT or rvM-PQS-1664(+)Mut-1 by anti-DDX60 was determined at 48 hours post infection by performing RNA immunoprecipitation (RIP) assays. (C and D) The DDX60 WT (Ctrl) and DDX60 knockout (DDX60-KO) Huh7 (B) and HepG2 (C) cells as well as DDX60-KO Huh7 and HepG2 cells transiently transfecting DDX60 were infected without or with rvM-PQS-1664(+)WT or rvM-PQS-1664(+)Mut at MOI 0.1., subsequently the ZsG fluorescence (green) was determined at 72 hours post infection. The mean of triplicate wells is represented by each point, with error bars indicating the SEM. The graphs presented here are representative of three independent experiments. **P < 0.01 by Student’s t test. (TIF) [file ppat.1013278.s012.tif]
